# Disentangling the link between zebrafish diet, gut microbiome succession, and *Mycobacterium chelonae* infection

**DOI:** 10.1186/s42523-023-00254-8

**Published:** 2023-08-10

**Authors:** Michael J. Sieler, Colleen E. Al-Samarrie, Kristin D. Kasschau, Zoltan M. Varga, Michael L. Kent, Thomas J. Sharpton

**Affiliations:** 1 Department of Microbiology, Oregon State University, Corvallis, OR 97330 USA; 2 Department of Biomedical Sciences, Oregon State University, Corvallis, OR 97330 USA; 3 Zebrafish International Resource Center, University of Oregon, Eugene, OR 97330 USA; 4 Department of Statistics, Oregon State University, Corvallis, OR 97330 USA

**Keywords:** Zebrafish, Gut microbiome, Development, Infection, Diet, Husbandry, *Mycobacterium chelonae*

## Abstract

**Background:**

Despite the long-established importance of zebrafish (*Danio rerio*) as a model organism and their increasing use in microbiome-targeted studies, relatively little is known about how husbandry practices involving diet impact the zebrafish gut microbiome. Given the microbiome’s important role in mediating host physiology and the potential for diet to drive variation in microbiome composition, we sought to clarify how three different dietary formulations that are commonly used in zebrafish facilities impact the gut microbiome. We compared the composition of gut microbiomes in approximately 60 AB line adult (129- and 214-day-old) zebrafish fed each diet throughout their lifespan.

**Results:**

Our analysis finds that diet has a substantial impact on the composition of the gut microbiome in adult fish, and that diet also impacts the developmental variation in the gut microbiome. We further evaluated how 214-day-old fish microbiome compositions respond to exposure of a common laboratory pathogen, *Mycobacterium chelonae*, and whether these responses differ as a function of diet. Our analysis finds that diet determines the manner in which the zebrafish gut microbiome responds to *M. chelonae* exposure, especially for moderate and low abundance taxa. Moreover, histopathological analysis finds that male fish fed different diets are differentially infected by *M. chelonae*.

**Conclusions:**

Overall, our results indicate that diet drives the successional development of the gut microbiome as well as its sensitivity to exogenous exposure. Consequently, investigators should carefully consider the role of diet in their microbiome zebrafish investigations, especially when integrating results across studies that vary by diet.

**Supplementary Information:**

The online version contains supplementary material available at 10.1186/s42523-023-00254-8.

## Introduction

In the effort to understand how the gut microbiome mediates vertebrate health, zebrafish (*Danio rerio*) have emerged as an important microbiome experimental model organism [1]. Despite the increasing use of zebrafish in microbiome research, key knowledge gaps remain about how different zebrafish husbandry practices, especially diet, influences microbiome composition [2, 3]. For example, in contrast to mice, zebrafish do not have a standard reference diet [4]. Instead, zebrafish research facilities vary by dietary husbandry practice, which can impact physiological and reproductive outcomes [5–7]. Diet plays an important role in shaping the composition of the gut microbiome in humans and across vertebrate and invertebrate animal models, such as mice and honeybees [8–13]. Therefore, we hypothesize that variation in dietary husbandry practice also impacts the composition of the zebrafish gut microbiome. Quantifying this association is important because it could explain why, despite the existence of a core gut microbiome, gut microbiome composition differs across research facilities [14, 15], improve efforts to integrate data across investigations, and clarify how dietary variation manifests as physiological variation.

Relatively little is known about how variation in dietary husbandry practice impacts the zebrafish gut microbiome. Prior studies that measured the impact of diet on the zebrafish gut microbiome have largely considered how substantial variation in specific macronutrients impacts the gut microbiome (e.g., high fat versus low fat diets) [6, 16–18]. This variation is not typically representative of the variation in nutrient content observed across standard dietary husbandry practices [4, 5]. Additionally, these studies have typically reared fish on a singular diet up to the point of experimentation, at which point fish are exposed to alternative diets. While insightful about acute effects, such experimental designs do not model the chronic dietary exposure that fish experience through husbandry. This prior work also does not typically consider how diet impacts the microbiome at different fish developmental periods, or whether dietary variation affects other characteristics of the gut microbiome, such as its sensitivity (i.e., changes in community composition) in response to exogenous agents (e.g., pathogens).

In this study, we sought to determine how the gut microbiome of early adult (129 days post fertilization, dpf) and fully mature (214 dpf) zebrafish is influenced by rearing them on different common facility diets. To do so, we reared fish throughout their lifespan on one of three different diets: fish were fed either (1) the Gemma (Skretting, Fontaine­les-Vervins, France) diet, which is a commercial feed widely used in zebrafish research facilities, (2) the ZIRC diet, a compound diet mixed and adopted by the Zebrafish International Research Center (ZIRC), which is one of the largest zebrafish stock centers in the world, or (3) a precisely defined laboratory grade diet developed by Watts [5]. Overall, these diets are relatively similar from a macronutrient perspective, though they differ by formulation, ingredient sourcing, manufacturing process details, and consequently also by exact nutritional content. (Table S4.1.1). In particular, we evaluated how the gut microbiome differed across these groups of fish as well as over development. We also determined if these differences in the microbiome link to variation in fish body size (weight and body condition score length normalized measure of weight). Lastly, we determined if fish fed different diets manifested differences in extraintestinal infection outcomes to one of the most common infection agents of zebrafish research facilities, *Mycobacterium chelonae* [19], as well as how the gut microbiome of fish fed different diets responds *M. chelonae* exposure.

## Results

**Fig. 1:**
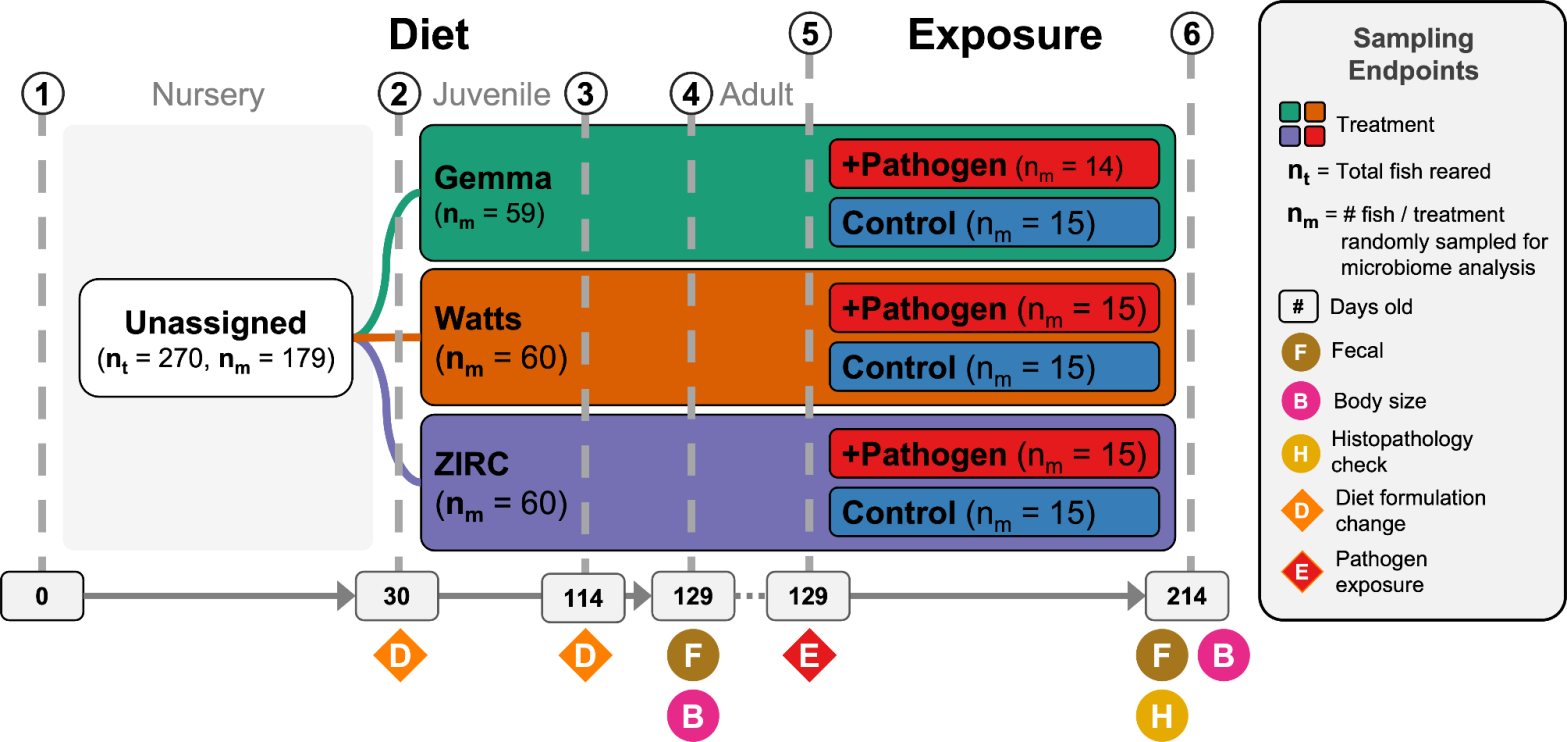
Experimental design showing treatments and husbandry events during the course of the study. Symbols indicate when an event occurred. (1) 270 fish were reared from 0 to 30 days post fertilization (dpf) on a nursery diet across 18 tanks (15 fish per tank). (2) At 30 dpf, fish were assigned one of three diets (e.g., Gemma, Watts, or ZIRC), and fed a juvenile formulation until 114 dpf. (3) At 114 dpf, fish were switched to an adult formulation of their respective diets. (4) At 129 dpf, body size measurements were conducted on all fish and fecal samples were collected from a random selection of five fish per tank (n = 90). (5) Afterwards, a cohort of fish from each diet were exposed to *Mycobacterium chelonae*. (6) Three months later when fish were 214 dpf, body size measurements were conducted on all fish and fecal samples were collected from a random selection of five fish per tank (n = 89). Histopathology check was conducted to assess infection burden on all fish

### Diet differentially influences physiology and gut microbiome at 129 days post fertilization

**Fig. 2:**
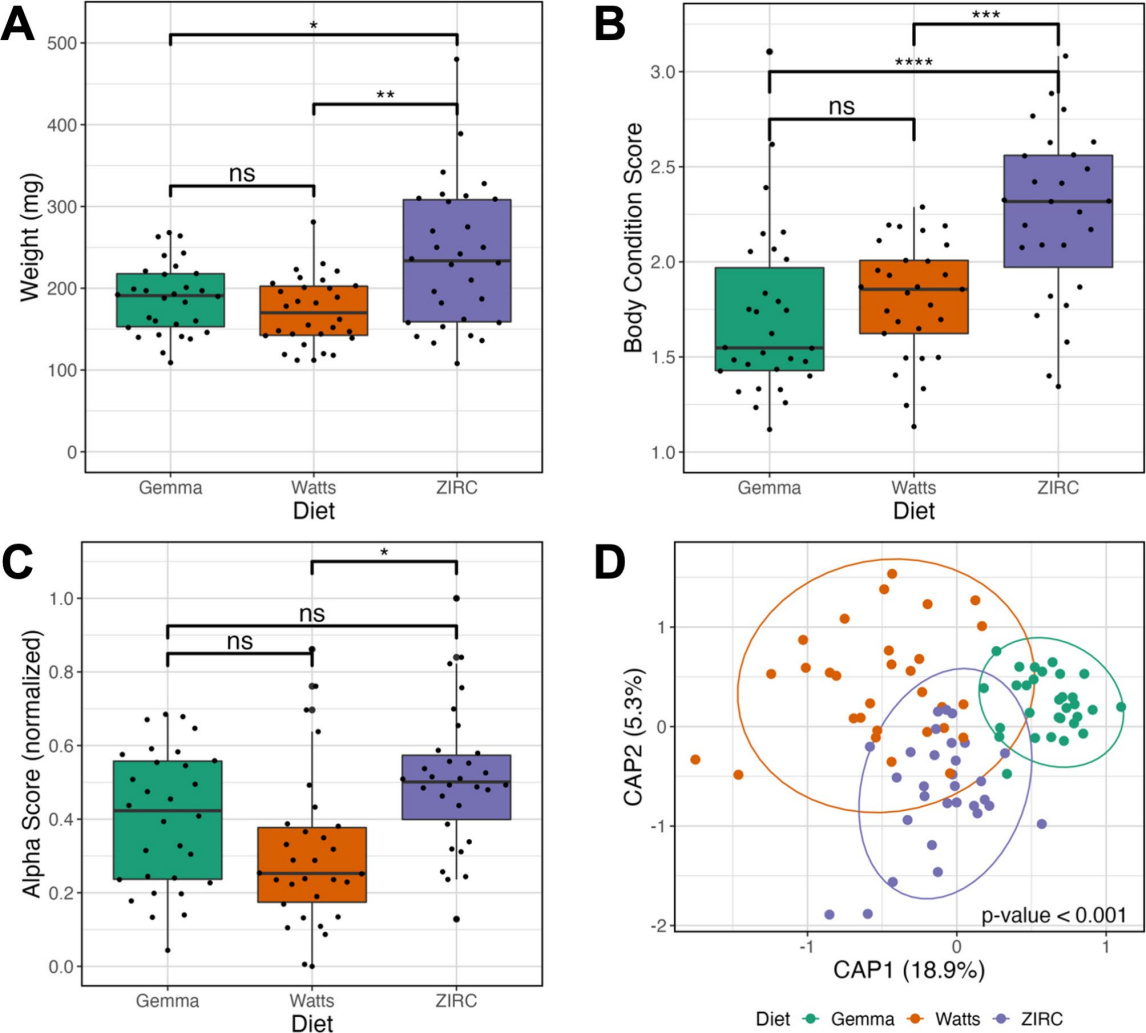
Effects of 129 days post fertilization (dpf) fish fed one of three diets (Gemma, Watts, or ZIRC) on physiology and microbiomes of zebrafish. **(A)** Weight of ZIRC-diet fed fish significantly differs from Watts- and Gemma-diet fed fish. Gemma- and Watts-diet fed fish do not differ from each other. **(B)** Body condition score is a length normalized measure of weight. Fish fed the ZIRC diet have significantly higher body condition scores from fish fed the Gemma and Watts diets. **(C)** Shannon Entropy of diversity shows that gut microbiome diversity significantly differs between Gemma- and Watts-diet fed fish, ZIRC- and Watts-diet fed fish, but not between Gemma- and ZIRC-diet fed fish. **(D)** Capscale ordination based on the Bray-Curtis dissimilarity of gut microbiome composition. The analysis shows that physiology and gut microbiome composition significantly differs between the diets. “ns” indicates not significantly different, *, **, *** indicates significant differences below the 0.05, 0.01, and 0.001 levels, respectively

To determine how common zebrafish diets differently impact fish size (weight and body condition score) and the gut microbiome, we reared 179 zebrafish that were assigned one of three diets from 30- to 214 days-post fertilization (dpf; Fig. 1): Gemma, Watts and ZIRC diets. Prior to diet assignment, fish were fed a nursery diet (see methods). At 129 dpf, we selected 89 individuals across these three cohorts and collected fecal samples from each fish for microbiome profiling prior to measuring their weight and body condition score (BCS). Wilcoxon Signed-Rank Tests found that diet and sex significantly associated with weight and BCS (Fig. 2A & B). Female fish had higher weight (Z = 1,530, P < 0.001; Table S1.1.2) and BCS (Z = 1,631, P < 0.001; Table S1.1.4) compared to males. Between the three diets, ZIRC-diet fed fish had the highest mean BCS compared to fish fed Gemma- (Z = 150, P < 0.001; Fig. 2B) and Watts-diet (Z = 197, P < 0.001; Table S1.1.3). Gemma- and Watts-diet fed fish did not significantly differ from one another in terms of weight and BCS. These results indicate that ZIRC-diet contributes to heavier fish compared to Gemma- and Watts-diet fed fish.

We next built generalized linear models (GLM) to determine if diet associated with variation in one of three measures of microbiome alpha-diversity: richness, Simpson’s Index, and Shannon Entropy. An ANOVA test of these GLMs revealed that alpha-diversity varies as a function of diet for all three measures of diversity we assessed (P < 0.05; Fig. 2C; Table S1.2.1). A post hoc Tukey test clarified that ZIRC- and Watts-diet fed fish exhibited significant differences in alpha-diversity as measured by richness and Shannon Entropy (P < 0.001, Table S1.2.2). Moreover, we observed significant differences in diversity between Gemma- and Watts-diet fed fish in terms of richness (P < 0.001; Table S1.2.2), and between Gemma- and ZIRC-diet fed fish when considering the Simpson’s Index (P < 0.001; Table S1.2.2). These results indicate that diet associates with fish gut microbiome diversity, and that diet may differentially impact rare and abundant microbial members of the gut.

To evaluate how diet associates with microbiome community composition, we quantified the Bray-Curtis, Canberra and Sørensen dissimilarity amongst all samples. We detected a significant clustering of microbial gut community composition based on diet as measured by all beta-diversity metrics (PERMANOVA, P < 0.05; Fig. 2D, Table S1.3.1). These results indicate that microbial communities of fish fed the same diet are more consistent in composition to one another than to fish fed other diets. Additionally, we assessed beta-dispersion, a measure of variance, in the gut microbiome community compositions for each diet group. We find the beta-dispersion levels were significantly different between the diet groups as measured by Bray-Curtis and Canberra metrics (P < 0.05; Table S1.4.1). Beta-dispersion levels were significantly reduced in Gemma-diet fed fish compared to Watts-diet fed fish when measured by Bray-Curtis metric, as well as significantly reduced compared to Watts- and ZIRC-diet fed fish when measured by Canberra metric (Table S1.4.1). These results indicate that Gemma-diet fed fish are more consistent in community composition than Watts- and ZIRC-diet fed fish at 129 dpf. Collectively, these results indicate that 129 dpf fish gut microbiome communities stratify by diet, but the composition of these microbial communities differ in consistency depending on diet.

Finally, to better understand the interactions between the diet and the members of the gut microbiome community, we quantified differential abundance using ANCOM-BC2. We observed 24 significantly abundant taxa at the genus level in at least one of the three diets (Table S1.5.1). Gemma-diet fed fish were enriched for *Chitinibacter* and were depleted of *Aeromonas* and *Flavobacterium*. Watts-diet fed fish enriched for *Flavobacterium*, *ZOR0006*, *Peptostreptococcus*, *Cetobacterium*, *Tabrizicola*, *Cellvibrio*, and unnamed genera of *Microscillaceae* and *Chitinibacteraceae*, and depleted of *Crenobacter* and a *Sutterellaceae* genus. ZIRC-diet fed fish enriched for *Cloacibacterium* and *Acinetobacter*, and depleted of *Fluviicola*. Many of these taxa are identified as common members of the zebrafish gut microbiome [14, 15]. These results indicate that diet differentially supports particular members of the zebrafish microbiome community.

### Diet impacts the successional development of the zebrafish gut microbiome

**Fig. 3:**
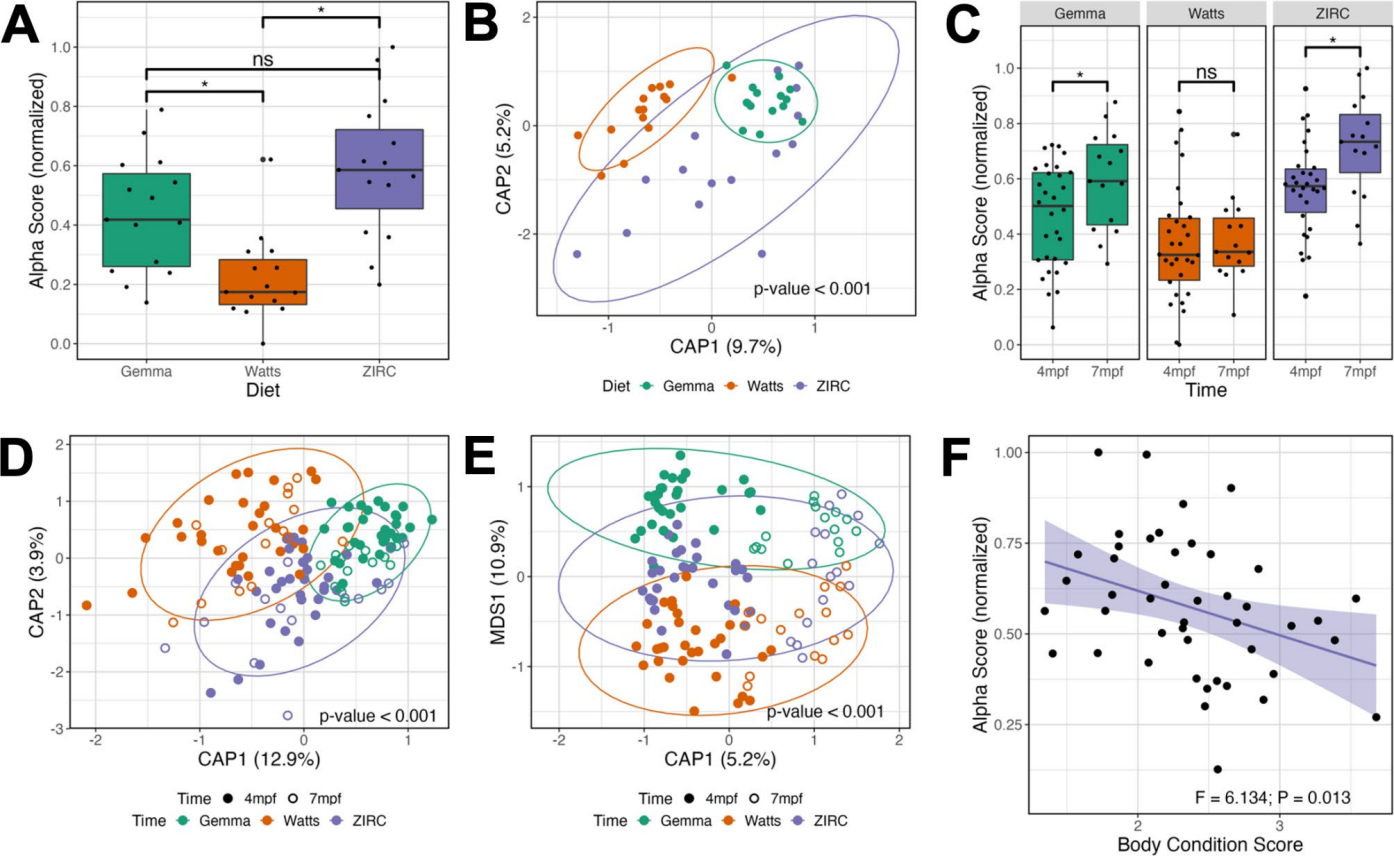
Development is associated with altered microbiome composition. **(A)** Shannon Entropy of diversity shows that gut microbiome diversity significantly differs between Watts-diet fed fish to fish fed the Gemma- and ZIRC-diets in 214 days post fertilization (dpf) zebrafish. **(B)** Capscale ordination based on the Bray-Curtis dissimilarity of gut microbiome composition in 214 dpf zebrafish. **(C)** Shannon Entropy for diversity shows microbial gut diversity increases with development in 129- to 214 dpf zebrafish fed the Gemma- and ZIRC-diets, but not Watts-diet fed fish. Capscale ordination of gut microbiome composition based on the **(D)** Bray-Curtis dissimilarity by diet and **(E)** Canberra measure by time. **(F)** Body condition score negatively associates with gut microbiome diversity as measured by Simpson’s Index across 129- and 214 dpf zebrafish fed the ZIRC diet. The analysis shows that fish size and gut microbiome composition significantly differs between the diets across development, and there may be diet-dependent link with physiology. A “ns” indicates not significantly different, “*” indicates significant differences below the 0.05 level

To determine how maintaining fish on different diets impacts the development of the gut microbiome, we continued to grow fish from the same diet cohorts until 214 days post fertilization (dpf; Fig. 1). Microbiome samples were collected from cohort members prior to quantification of fish weight and body condition score. To determine the effect of diet on the body condition score and the gut microbiome of 214 dpf fish, we conducted the same analyses as we applied to the 129 dpf fish. At 214 dpf, we find body condition score is significantly associated with diet (P < 0.05; Table S2.1.3.1). Additionally, linear regression analyses revealed statistically significant main effects of diet on gut microbiome alpha- and beta-diversity for all metrics we considered (P < 0.05; Fig. 3A & B, Table S2.1.3.2-3). Furthermore, an ANOVA test of beta dispersion found significant levels of dispersion as measured by the Canberra metric (P < 0.05; Table S2.1.3.4), but the Bray-Curtis and Sørensen metrics did not reach our threshold for significance (P > 0.05; Table S2.1.3.4). These results demonstrate that diet impacts the physiology and gut microbiome of 214 dpf fish.

Next, we compared our results between the 129- and 214 dpf fish to determine how diet impacts the successional development of the gut microbiome. Linear regression revealed microbial gut alpha-diversity was significantly associated with the main effect of time (P < 0.05; Table S2.2.1) for each diversity metric. However, we did not find a diet dependent effect on time for any alpha-diversity metric we assessed (P > 0.05; Table S2.2.1). A post hoc Tukey test clarified that microbiome diversity was significantly different between 129- and 214 dpf Gemma- and ZIRC-diet fed fish as measured by the Shannon and Simpson’s alpha-diversity metrics (P < 0.05; Fig. 3C, Table S2.2.2), but we did not find a statistically significant association between 129- and 214 dpf Watts-diet fed fish with any alpha-diversity metric (P > 0.05; Table S2.2.2). These results indicate that the alpha-diversity of the gut microbiome of Watts-diet fed fish were temporally stable, while Gemma- and ZIRC-diet fed fish diversified over time in diet-consistent ways.

A PERMANOVA test of the 129- and 214 dpf samples using the Bray-Curtis dissimilarity metric revealed that community composition was best explained by diet (P < 0.05; Fig. 3D, Table S2.3.1), but an analysis using the Canberra measure found that variation in microbiome composition was best explained by time (P < 0.05; Fig. 3E, Table S2.3.2). Given how these metrics weigh the importance of abundant versus rarer taxa, respectively, these results indicate that abundant members of the microbiome community are more sensitive (i.e., exhibit greater amounts of change) to the effects of diet, while rarer community members are sensitive to the effects of time. Moreover, we found beta-dispersion levels were significantly elevated between 129- and 214 dpf Gemma-diet fish when considering the Bray-Curtis and Sørensen metrics, in Watts-diet fed fish when considering the Canberra and Sørensen metrics, and in ZIRC-diet fed fish across all three beta-diversity metrics (P < 0.05; Table S2.4.1-3). These results indicate that abundant and rarer gut microbiome community members were differentially impacted by the effects of time depending on diet. Collectively, these results indicate that diet can have a substantial impact on how the gut microbiome successionally develops in zebrafish.

Differential abundance analysis revealed taxa that were significantly associated with the effects of time and diet in one of the diet groups (Table S2.5.1). Across all three diets, the taxa that were more abundant included *Fluviicola*, *Macellibacteroides*, *Bacteroides* and an unnamed genus in the *Barnesiellaceae* family were, while taxa that were less abundant included *Phreatobacter* and *Flavobacterium*. These results indicate that irrespective of diet, the abundances of taxa change over the course of zebrafish development. We also measured how taxon abundance changed over time within each diet (Figure S2.5.2–46.2.5). The Gemma-diet fed fish uniquely enriched for *Exiguobacterium* (Table S2.5.2). *Exiguobacterium* are gram-positive facultative anaerobes in the phylum Bacillota, and are linked to fatty acid metabolism in zebrafish [20, 21]. The Watts-diet fed fish were uniquely depleted of *Gemmobacter* (Table S2.5.3). Previous work has found that *Gemmobacter* has a positive association with parasite exposure in infected zebrafish [22, 23]. The ZIRC-diet fed fish were uniquely enriched for *Pseudomonas* and *Haliscomenobacter* (Table S2.5.4). *Pseudomonas* is a common member of the gut microbiome and associated with fatty acid metabolism in zebrafish [20]. Less is known about the *Haliscomenobacter* genus, but an analysis of its genome revealed it is an aerobic chemoorganotroph found in aquatic systems [24]. Together, these results indicate that particular members of the gut microbiome associate with diet and zebrafish development.

To determine if fish size associated with diet across zebrafish development, we used Wilcoxon Signed-Ranks Tests to identify parameters that best explained the variation in body condition score (BCS) between 129- and 214 dpf fish. At 129 dpf, the BCS significantly differed between fish fed different diets (P < 0.05; Table S2.1.1). However, we did not find that BCS of fish were impacted by time (P > 0.05; Table S2.1.1). These results indicate that while fish differ in BCS between diets at 214 dpf, their weight and length grow proportionally at a similar rate from 129- and 214 dpf. Interestingly, we observed a significant negative association of BCS and microbial gut diversity uniquely in fish fed the ZIRC diet as measured by Shannon Entropy and Simpson’s Index (P < 0.05; Fig. 3F, Table S2.1.2.1). This result indicates that fish gut microbiomes with higher body condition scores are lower in diversity compared to fish with lower body condition scores. For Canberra and Sørensen beta-diversity metrics, there were significant main effects of body condition score, and significant interaction effects between BCS and diet (P < 0.05; Table S2.1.2.2). However, the model coefficient for the effect of body condition score and its interaction with diet is far smaller than the coefficient for the effect of diet (Table S2.1.2.2). We did not find a significant association between BCS and specific taxon abundance (Table S2.1.2.2). Collectively, these results indicate that while the gut microbiome’s composition associates with BCS, the effect of diet on the gut microbiome is much stronger.

### Fish fed different diets are differentially infected by ***Mycobacterium chelonae***

**Fig. 4:**
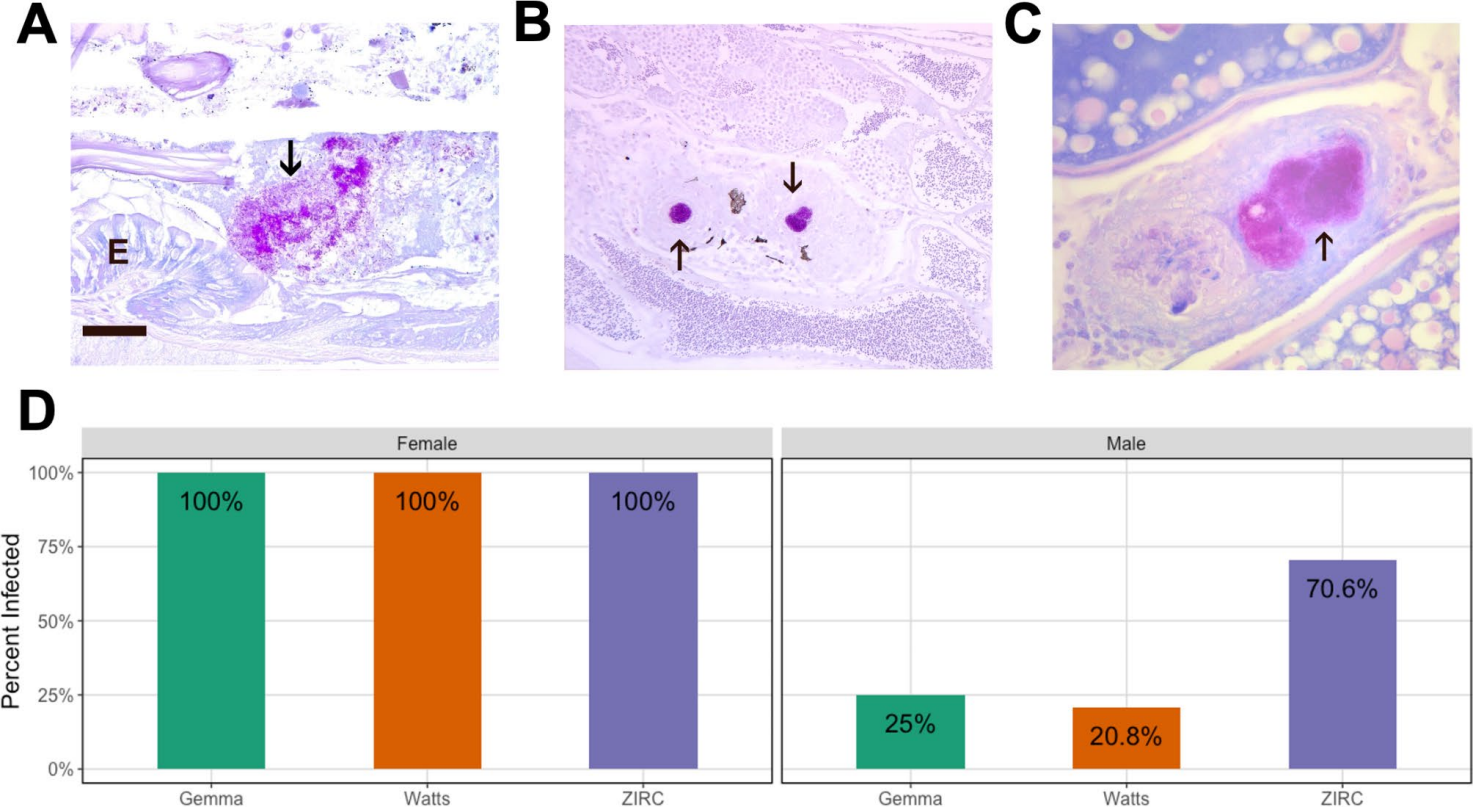
Histologic sections stained with Kinyon’s acid fast stain in zebrafish exposed to *Mycobacterium Chelonae* examined at 15 week post exposure. Arrow = acid fast bacteria. **(A)** Bacteria in intestinal lumen (E = intestinal epithelium, Bar = 25 μm). **(B)** Testis with two granulomas with acid fast bacteria. **(C)** Ovary with two granulomas, one containing abundant acid fast bacteria. **(D)** Infection outcome analysis of male and female fish injected with *M. chelonae* (n = 66). All exposed female fish were positive for infections, but male fish differed in infection outcomes depending on diet

Next, we sought to determine how zebrafish respond to the common pathogen of zebrafish, *Mycobacterium chelonae*. Mycobacteria has been reported in zebrafish from about 40% of research facilities and is a major driver of mortality across research facilities [25]. *Mycobacterium chelonae* infection is usually only diagnosed by histology, and hence is only diagnosed to the genus level based on the presence of acid-fast bacteria. When species identifications are made using molecular methods, the identification is most frequently *M. chelonae* [26]. It is hypothesized to be introduced through diet early in life [25, 27, 28]. *M. chelonae* forms granulomas coelomic organs, swim bladder and kidney, and in many cases, it ultimately causes death. Despite the extensive research into the pathogenesis of *M. chelonae*, very little is known about the factors that determine infection outcomes. Diet has been hypothesized to influence infections and may be a currently cryptic determinant of *M. chelonae* infection [28]. To clarify whether diet affects *M. chelonae* infection, we injected *M. chelonae* into the coelomic cavities of fish from each diet cohort at 129 dpf following fecal collection. These *M. chelonae* injected fish comprised the pathogen exposure cohort for this experiment, which we compared to the remaining, unexposed cohort of fish in our subsequent microbiome analyses. At 214 dpf, we performed a histopathological analysis of intestinal tissue to assess infection rate, and measured body condition score. These 214 dpf fish that were exposed to *M. chelonae* were utilized to determine if infection rates differ across fish as a function of diet.

We first evaluated whether diet impacted infection outcomes, as determined by histological confirmation of infection 3.5 months following pathogen injection. We conducted a Chi-Square test to compare the infection count between fish fed the three diets. The results showed that there was a statistically significant difference in infection rates between the groups (X2 = 11.519, df = 2, N = 66, P < 0.05; Table S3.1.1). Across all three diets, all females had infected ovaries (Fig. 4C), indicating there is no diet-driven difference in infection rates for female fish. As a result, we verified that the differential infection rates across diet groups was driven by male fish in a follow-up Chi-Square test using only male fish. This analysis confirms that infection rates are statistically different between male fish fed the ZIRC diet as compared to male fish fed either the Gemma or Watts diets (X2 = 11.556, df = 2, N = 53, P < 0.05; Fig. 4D, Table S3.1.2). Because we obtained infection data from all injected fish and corresponding controls reared in our study, whereas we produced microbiome data from only a subset of these fish, we also determined whether this pattern holds across the subset of male fish for which we also have microbiome data. This Chi-Square test finds no significant effect, (X2 = 4.069, df = 2, N = 44, P > 0.05; Table S3.1.3.1-2), likely due to being underpowered to detect infection rate differences on this relatively small subset of the data. Infections in males included the testis (Fig. 4B), coelomic cavity, swim bladder and kidney. We observed colonization of the intestinal lumen by acid fast bacteria across all three diets in both male and female fish (Fig. 4A). A linear regression did not find evidence of an association between extraintestinal infection of zebrafish and body condition score across all fish (P > 0.05; Table S3.1.4.1), even when considering sex (P > 0.05; Table S3.1.4.2). Taken together, these results indicate that the diet impacts *M. chelonae* infection outcomes in zebrafish, but not in a way that manifests as differences in body condition score (i.e., fish size).

### Diet influences gut microbiome’s sensitivity to pathogen exposure

**Fig. 5:**
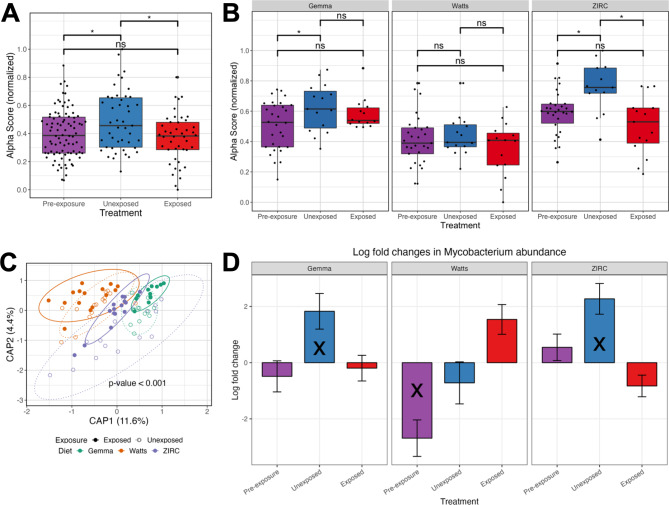
Exposure to *Mycobacterium chelonae* inhibits diversification of gut microbiome. **(A)** Shannon Index for diversity of pre-exposed 129 days post fertilization (dpf), 214 dpf exposed and unexposed fish, and **(B)** for exposure groups within each diet. Capscale ordination based on the Bray-Curtis dissimilarity of gut microbiome composition of fish by **(C)** diet. **(D)** Log fold change of *Mycobacterium* of pre-exposed, exposed and unexposed fish within each diet as calculated by ANCOM-BC. Values are in reference to exposed fish within each diet. The analysis shows gut microbiome’s sensitivity to pathogen exposure is linked to diet, but *Mycobacterium*’s abundance is diet-dependent. A “ns” indicates not significantly different, and * indicates significant differences below the 0.05. An “X” indicates a group is significantly differentially abundant compared to the exposed treatment reference group

Lastly, we sought to determine whether the gut microbiome changes in response to *Mycobacterium chelonae* infection. *M. chelonae* infections can introduce inconsistencies in study outcomes, but the impacts on the gut microbiome are not known [25]. Prior to pathogen infection at 129 dpf, we collected fecal samples of a subset of fish for microbiome analysis. At 214 dpf, we collected fecal samples from control and pathogen exposed fish. The 129- and 214 dpf fecal samples were then measured for microbial gut diversity, composition, and taxon abundance. We next built generalized linear models (GLM) to determine if extraintestinal infection as a function of diet associated with microbial diversity and composition measures. We did not observe any significant associations between extraintestinal infection and any of the gut microbiome diversity and composition measures (P > 0.05; Table S3.1.5-6), likely because we were under powered to detect a difference. While not all fish that were injected with *M. chelonae* manifested evidence of an infection at 214 pdf, all of these fish were exposed to the pathogen. Since gut microbiomes in zebrafish diversify in response to pathogen exposure, we next asked how exposure to *M. chelonae* affects the zebrafish gut microbiome and whether these effects differ across diets [22]. Comparing exposed to unexposed fish found that microbial gut diversity significantly differs between exposure groups as measured by richness and Shannon Entropy alpha-diversity metrics (P < 0.05; Fig. 5A, Table S3.2.1). That said, based on linear regression, the impact of exposure on the gut microbiome alpha-diversity does not appear to differ as a function of diet, as the interaction term for these covariates did not yield a significant effect (P > 0.05; Table S3.2.1). Furthermore, we used a post hoc Tukey test to clarify whether microbial gut diversity of fish differed between exposure groups by diet. Unique to ZIRC-diet fed fish, we observed microbiome diversity differed in unexposed controls compared to exposed fish as measured by all alpha-diversity metrics (P < 0.05; Fig. 5B, Table S3.2.2). Watts-diet fed fish differed in unexposed controls compared to exposed fish in terms of richness (P < 0.05, Table S3.2.2). These results suggest that the gut microbiome diversity of ZIRC-diet fed fish, and to some extent Watts-diet fed fish, are more impacted by the effects of *M. chelonae* exposure, but Gemma-diet fed fish are less impacted by pathogen exposure. While the gut microbiomes are impacted by the effects of pathogen exposure, we find the statistical effect of diet shaping the gut microbiome is an order of magnitude greater across all alpha-diversity metrics (P < 0.05, Table S3.2.1). Collectively, these results indicate that gut microbiome diversity is sensitive to *M. chelonae* exposure, but diet is the primary driver of gut microbiome diversity.

Next, we evaluated how pathogen exposure influenced microbial community composition across fish fed each diet. For each beta-diversity metric considered, PERMANOVA tests found that the main effects of diet and pathogen exposure significantly explained the variation in microbiome composition, but that the main effect of diet was consistently larger than the effect of exposure (P < 0.05; Fig. 5C, Table S3.3.1). Furthermore, a PERMANOVA test found that the model coefficient effect for the interaction of diet and pathogen exposure was statistically significant when considering Canberra and Sørensen beta-diversity metrics, however this effect was marginal as compared to the aforementioned main effects. Moreover, a pairwise analysis of beta-dispersion did not find significant levels of dispersion between exposed and unexposed fish within each diet (P > 0.05; Table S3.4.1-3). These results indicate that exposure to *M. chelonae* did not affect dispersion of the gut microbiome communities. Collectively, these results indicate that the gut microbiome is impacted by pathogen exposure, but that dietary effects tend to overwhelm the effects of pathogen exposure.

We also observed several microbiota that stratified exposed and unexposed groups of fish in both diet-robust and diet-dependent manners. Unexposed Gemma-diet fed fish were enriched for *Macellibacteroides* and *Aurantisolimonas* (Table S3.5.2), unexposed Watts-diet fed fish were enriched for an unnamed genus of *Barnesiellaceae*, *Fluviicola*, *Paucibacter*, and *Brevibacterium* (Table S3.5.3), and unexposed ZIRC-diet fed fish were enriched for *Macellibacteroides*, *Bacteroides*, *Mycobacterium* and unnamed genera of *Barnesiellaceae* and *Sutterelaceae* (Table S3.5.4). Across all the diets, the taxa that were more abundant in unexposed, control fish included *Macellibecateroides*, *Fluviicola*, *Bacteroides*, *Aurantisolimonas*, *Cerasicoccus*, and three unnamed genera of *Barnesiellaceae*, *Commonadaceae*, and *Sutterellaceae*. *Plesiomonas* were more abundant in exposed fish compared to controls (Table S3.5.1). These results indicate that pathogen exposure impacts the abundance of certain taxa within and across the diets. Next, to see if *Mycobacterium* species abundance differed from background, pre-exposure levels we compared *Mycobacterium* abundance between pre-exposure and unexposed control fish to that of exposed fish within each diet. Unexposed Gemma- and ZIRC-diet fed fish had significantly higher abundances of *Mycobacterium* to exposed fish (Q < 0.05; Fig. 5D, Table S3.5.5). Pre-exposed Watts-diet fed fish had significantly more *Mycobacterium* compared to pre-exposed Watts-diet fish, but they did not differ significantly from unexposed Watt-diet control fish. These results indicate that the abundance of taxa from the genus *Mycobacterium* changes in response to exposure to a pathogenic species in a diet-dependent manner.

## Discussion

Zebrafish are an important emerging model organism for understanding the microbiome. Yet, there is little consistency across studies in terms of the husbandry practices used to conduct zebrafish microbiome experiments, especially in terms of diet. This lack of consistency likely stems from a dearth of knowledge about how different standard zebrafish diets impact study outcomes, both in terms of the gut microbiome’s composition and the physiological endpoints of the host. Our study offers critical insight into how three standard zebrafish dietary formulations impacts these outcomes, finding that the zebrafish gut microbiome’s development and response to pathogen exposure is more impacted by diet. These observations help clarify inconsistencies across studies, underscore the importance of considering diet when integrating data across investigations, and inform on efforts to develop standard approaches in zebrafish microbiome research.

We found that diet had a substantial impact on the structure of the gut microbiome in adult zebrafish. Previous research has found that diets with varying compositions of key macronutrients (e.g., protein, lipids, and fiber content) impacts zebrafish physiology and the gut microbiome [5, 16–18, 29–32]. Moreover, diet’s effect on restructuring the host’s gut microbiome has been observed across an evolutionarily diverse array of vertebrate and invertebrate animal hosts [8, 9, 11, 12, 33]. However, the nutritional compositions used in these prior studies tend to vary considerably. In particular, the feeds our study considered are far more consistent in their composition than the diets that are typically included in studies of the effect of diet on the gut microbiome (e.g., high-fat v. low-fat diets). Moreover, a unique strength of our study is that fish were fed the same diets over the vast majority of their lifespan (30 to 214 dpf), which is more consistent with a standard husbandry approach that maintains fish on a specific diet than the relatively short-term exposures to different types of diet that are typically employed in related research. Because of these features of our experimental design, our work provides important clarity into how seemingly subtle differences in husbandry practice can result in substantial differences in the composition of the adult zebrafish gut microbiome.

We also found that diet impacts the developmental variation in the gut microbiome. Prior work investigating the successional development of the zebrafish gut microbiome has had inconsistent results; our efforts indicate that these inconsistencies may be attributable to the different diets utilized in these prior studies [18, 29, 30, 32]. For instance, Stephens et al. used a variety of live and dry food diets and found that juvenile zebrafish gut microbiomes were highly diverse but declined with age [30], while Wong et al. found opposite results for juvenile zebrafish that were fed defined diets [18]. Furthermore, prior work indicates that early life variability of the gut microbiome could be a result of husbandry choices involving diet [29, 31, 32]. Despite differences in study duration, we find congruent trends in gut microbiome diversity to these previous studies when comparing sampling time points within similar developmental periods. However, comparing our results to prior studies is challenging because of differences in sampling time points, varied diets used, and undisclosed diet information. It is worth nothing that while our fish were fed the same diet from 30 days onward, at 114 dpf fish in our study were switched from a juvenile formulation to an adult formulation of their respective diets. These formulations differed slightly in some diets (e.g., Gemma and Watts), but in others more substantially (e.g., ZIRC). These differences in formulation may contribute to the variability we observed in the gut microbiome between diets across zebrafish development. Despite these limitations, we found adult zebrafish fed diets of similar nutritional composition manifest distinct gut microbiome successional patterns in community compositions across adulthood. Future work should seek consistency in diet formulations and increase sampling time points throughout zebrafish development to further clarify the successional development of zebrafish gut microbiomes.

Finally, we observed that the gut microbiome of zebrafish were sensitive to pathogen exposure, but diet was the main driver of gut microbiome structure. We ensured all fish were exposed to the pathogen by injecting *Mycobacterium chelonae* into the coelomic cavities of the fish at 129 dpf. We found that presence of infection was not sufficient to explain associations with microbiome diversity or community composition, which is likely due to being under powered to detect them. Additionally, we did not observe an association between infection outcomes and body condition score (i.e., fish size), which aligns with prior work that did not observe effects of *M. chelonae* infection on fish size in a larger cohort of zebrafish [34]. Furthermore, we found infection by diet interactions on a larger number of individuals who were assessed for histopathology, but not with the subset of fish sampled for microbiome analysis. This indicates that having a sufficiently large sample size is important for observing infection effects on the gut microbiome. However, we found that gut microbiome diversification did not change after exposure to *M. chelonae*, except in ZIRC-diet fed fish relative to their unexposed controls. We did find that fish fed different diets show differential infection outcomes and microbiome sensitivities to pathogen exposure, which may indicate that the diet driven microbiome differences are a defining factor in infection outcomes. Alternatively, certain diet-driven microbiome compositions may be more susceptible to perturbation, and thus may be more likely to yield dysbiosis following pathogen exposure compared to fish fed other diets. For husbandry purposes, these observations are important considerations regarding long-term health management of fish, especially given that mycobacteriosis is the second most common infection in zebrafish research facilities (over 35% of all facilities). Additionally, *M. chelonae* is thought to drive non-protocol induced variation in zebrafish studies possibly as a result of dysbiosis, which can undercut experimental conclusions [35].

Our results contrast our prior work that found exposure to an intestinal helminth was associated with an increase in microbiome diversity [22]. One possible explanation for this discrepancy is our prior study investigated an intestinal helminth which may have different impacts on the gut microbiome associated with differences in intestinal lesion to that of a pathogenic bacterial species. For example, the nematode *Pseudocapillaria tomentosa* penetrates the intestinal epithelium and causes profound pathologic changes [22], whereas disease caused by *Mycobacterium* species in zebrafish are characterized by extraintestinal infections and lesions [25]. *Mycobacterium* spp. in zebrafish are hypothesized to be introduced early in life through ingestion, including diet [28, 36], while fish in our study were exposed by injection into their coelomic cavities at adulthood when their gut microbiomes have been firmly established. Priority effects may have hindered the injected species of *Mycobacterium* from more substantially altering the gut microbiome at adulthood than if it had been introduced through a natural route during early life microbiome assembly [28, 36, 37]. Future work should consider using a natural mode of infection and exposing fish to a variety of pathogens to elucidate the gut microbiome’s role in mediating pathogen exposure. Furthermore, because we found that the effect of diet was far greater than pathogen exposure on shaping the gut microbiome, future studies must consider diet effects, as they may overwhelm infection effects.

In conclusion, we found diet is one of the most important factors driving variation in the zebrafish gut microbiome. Unlike prior studies, including the extensive research conducted in mammalian models, that have evaluated dietary effects on the gut microbiome using diets that fundamentally differ in macronutrient composition, our work reveals that even relatively consistent diets that are commonly selected as normal husbandry practices elicit these large impacts on microbiome composition. While the zebrafish gut microbiome differs taxonomically from other animal systems, there is a substantial amount of shared functional capacity between zebrafish and mammalian gut microbiomes [38]. Consequently, the taxa-specific associations we found here may not directly translate to other animal systems, but the interactions between the microbiome, diet and pathogen exposure may be similar. Notably, our work used fecal samples, which may not appropriately reflect all members of the zebrafish gut microbiome, in particular mucosa associated taxa. Therefore, mucosal populations of microbiota may manifest different patterns with response to diet compared to taxa we observed in the fecal microbiota [39]. Further complicating investigations of diet’s effect on the gut microbiome are inconsistencies within diets introduced through the manufacturing process that vary in ingredient sourcing and nutrient profile between batches [4]. Future work should illuminate the underlying mechanisms of the diet’s influence on zebrafish development, gut microbiome structure and the microbiome’s sensitivity to pathogen exposure. Collectively, our study demonstrates that investigators should carefully consider the role of diet in their microbiome-targeted zebrafish investigations, especially when integrating results across studies that vary by diet.

## Conclusions

Collectively, our study demonstrates the effect of commonly used laboratory diets on the gut microbiome of zebrafish. We reared zebrafish across their lifespan on three commonly used diets and analyzed the gut microbiome of juvenile and adult fish. Our findings demonstrate that diet impacts the developmental trajectories of the zebrafish gut microbiome, even with similar nutritional compositions. Additionally, diets were found to differentially sensitize the gut microbiome to pathogen exposure, and in the case of male fish result in different rates of infection. These results have important implications for the practice of zebrafish husbandry and the selection of diets in microbiome studies. Our findings will also contribute to ongoing discussions about standardizing husbandry practices, including diet, in the zebrafish research community.

## Methods

### Fish Husbandry

A total of 270 30 days post fertilization (dpf) AB line zebrafish were randomly divided into eighteen 2.8 L tanks (15 fish/tank) on a single pass flow-system tanks (15 fish/tank). During the experiment, temperature was recorded daily and ranged from 25.5 to 28.3 °C, with the exception of two isolated overnight temperature drops below that range due to two separate power loss events that affected the source water sump heater. All other water conditions were monitored weekly, pH ranged from 7.0 to 7.6, total ammonia ranged from 0 to 0.25 ppm (measured with pH and ammonia API test kits; Mars Fishcare North America Inc. Chalfont, PA), and conductivity ranged from 109 − 166 microsiemens. Light in the vivarium was provided for 14 h/day. One plastic aquatic plant piece approximately 6 inch in length was added to each tank for enrichment when fish were 129 dpf. A stock of similarly aged Casper line fish were maintained for the duration of the experiment, with a third of the stock being maintained on each of the diet regimens matching the AB line zebrafish. These fish served as filler fish and were added to the tanks after each histological sampling time point to maintain the 15 fish/tank ratio required to maintain the prescribed food-to-fish density per feeding as well as mitigate social stress effects on the fish. Casper fish were not sampled for microbiome or infection analyses.

### Diets

Fish were all fed the same nursery diet until 30 dpf, a combination of paramecia, brine shrimp, and the ZIRC Nursery Mix: Zeigler AP Larval Diet (Ziegler Bros Inc., Gardners, PA) and freeze dried rotifers. Fish were then transferred to the OSU facility and assigned randomly to one of three juvenile diets: Gemma Micro 150/300 (Skretting, Fontaine­les-Vervins, France), Watts High-Fat Juvenile Mix, or ZIRC Juvenile Mix, twice daily (9 AM and 3 PM local time) until 60 dpf. From 60 dpf onward, OSU fish were not fed on weekends and 1-day holidays as per the facility institutional animal care and use protocol. The total quantity fed daily was 3% fish body weight. This continued until fish were 114 dpf and then they were transitioned to the adult version of their previously assigned juvenile diet: Gemma Micro 500 (Skretting, Fontaine­les-Vervins, France), Watts Low-Fat Adult Mix, or ZIRC Adult Mix, twice daily (9 AM and 3 PM local time), except weekends and 1-day holidays. The total quantity fed daily was 3% fish body weight. The prescribed amounts of each diet regiment, for both the juvenile and adult diets were delivered by 3D printed spoons specific to the diet and stage of life. These spoons were paired with conical tubes retrofitted with leveling wires to ensure consistent feeding volumes as prescribed. All fish were only fed once, in the afternoons, on sampling days. Proximate analysis of diets used in the study can be found in the supplementary material.

### Diet and Pathogen exposure

Each of the eighteen tanks was assigned one of the three diet regimens: Gemma, Watts, or ZIRC. There were three tank replicates per diet regimens for a total of nine tanks that were exposed to *M. chelonae* via intraperitoneal injection (3 tanks/diet with 15 fish/tank). The remaining nine tanks were similarly assigned to diet regimens and were exposed to a sterile 1X-phosphate buffered saline (PBS) solution via intraperitoneal injection. Each fish was injected with 10 µLof either the *M. chelonae* inoculum or saline solution. The injections were completed over the course of two days and the *M. chelonae* inoculum was prepared as a 0.5 McFarland each day with a target dose/fish of 5 × 10^4^ viable bacteria/fish. This target dose was chosen as we have found that it induces a higher prevalence of *M. chelonae* in zebrafish with minimal mortality [34, 40, 41].

Day 1 *M. chelonae* inoculum was afterwards determined by plating to be 3.1 × 10^3 dose per fish, while Day 2 *M. chelonae* inoculum was determined by plating to be 1.0 × 10^5 dose per fish. For ZIRC and Gemma, two tanks for ZIRC fish were injected on Day 1, and 1 tank on Day 2. For Watts, one tank was injected on Day 1 (low dose) and 2 tanks were injected on Day 2 (high dose). No significant difference was observed in prevalence, so further analyses treated the exposed fish with in each diet group together.

Low and high dose across tanks:

- Gemma:
  - Low: Tank 14 and 35.
  - High: Tank 26.
- Watts.
  - Low: Tank 6.
  - High: Tank 12 and 33.
- ZIRC
  - Low: Tank 7 and 10.
  - High: Tank 4.

### Growth parameters and sex determination

Growth and sex parameters were collected when fish were 129–130 and 213–214 dpf for interfacility comparison. Sex was determined by gross differences in morphology and confirmed by histology for all samples collected for disease severity evaluation. Following overnight fecal collection, individual fish would be placed in a pre-anesthetic solution of 50 ppm MS-222 prepared with Tricaine-S (Western Chemical Inc., Ferndale, WA; a subsidiary of Aquatic Life Sciences Inc.) briefly before being transferred to a 150 ppm MS-222 anesthetic solution in a Petri dish on centimeter grid paper to be photographed. Fish were photographed when immobile but still upright. Standard length and width were evaluated via photographs taken with an iPhone (Apple Inc., Cupertino, CA) and analyzed with ImageJ software (https://imagej.net). Weight was obtained while the fish was still under the effects of anesthesia by transferring them from the photography Petri dish to a Petri dish on a scale with a volume of tared fish water, with excess water was removed. Body condition score is a length normalized metric of weight (for equation, see Methods) and serves as a general indicator of health in zebrafish and was calculated using the following equation:

- BCS = Weight (mg)/Length (mm)^3^ × 100.

### Histopathology

Fish were euthanized by hypothermia preserved in Dietrich’s solution, processed, and slides stained with Kinyoun’s acid-fast [42]. Fish were processed into mid-sagittal sections as previously described [43]. Infection in fish were scored as positive when acid fast bacilli were observed in extra-intestinal organs [43]. A Chi-square test was used to compare positive and negative infections between fish fed each diet.

### Fecal Collection

Five fish from each tank at 129- and 214-days post fertilization sampling time points were randomly selected for fecal sampling. Fecal material was collected from individual fish at the same sample intervals as outlined for the growth parameters. Fecal collection was set up the day before growth parameter sampling. Fish were transferred to 1.4 L tanks (1 fish/tank) containing ~ 0.4 L of fish water at least 30 min after the last feeding of the day. Fish were left to defecate overnight and all fecal material was collected from each tank the following morning in a 1.5ml microcentrifuge tube. Fecal samples were immediately spun at 10k rpm for 2 min, excess tank water was removed, and samples were snap frozen on dry ice and stored at -80 ˚C until processing.

### 16 S sequencing

Microbial DNA was extracted from zebrafish fecal samples and 16 S rRNA gene sequence libraries were produced and analyzed following established approaches [44]. Briefly, the DNeasy PowerSoil Pro DNA kits (Qiagen) were used to extract and purify DNA. The V4 region of the 16 S rRNA gene was PCR amplified using the Earth Microbiome Project 16 S index primers and protocols (Walters et al., 2016). PCR products were visualized on a 1.5% agarose gel and quantified on a Qubit 2.0 (Thermofisher Scientific) using the Qubit dsDNA HS Assay. One hundred nanograms of PCR product for each DNA sample was pooled and cleaned using the QIAquick PCR Purification Kit (Qiagen). The quality of the pooled library was verified on the Agilent TapeStation 4200. The prepared library was submitted to the Oregon State University Center for Quantitative Life Sciences (CQLS) for 300 bp paired-end sequencing on an Illumina MiSeq System (RRID:SCR_016379).

### Statistical analysis

All microbiome DNA sequence analyses and visualizations were conducted in R (v 4.2.1) [45]. Fastq files were processed in using the DADA2 R package (v 1.18.0) [46]. Briefly, forward and reverse reads were trimmed at 250 and 225 bp, respectively, subsequently merged into contigs, and subject to amplicon sequence variant (ASV) identification. ASVs unannotated at the Phylum level were removed to result in 2029 remaining detected ASVs. We used Wilcoxon Signed-Ranks Tests to identify parameters that best explained the variation in weight and body condition scores. Alpha-diversity was calculated using the estimate_richness function (Phyloseq v 1.38.0) and transformed using Tukey’s Ladder of Powers using methods described previously [44]. After transformation, scores were normalized from 0 to 1 by dividing each score by the maximum value, which allowed us to compare results across alpha-diversity metrics using general linear models (GLMs). Post hoc Tukey Tests evaluated pairwise comparisons of models using multcomp (v1.4-2) glht function [47]. We corrected for multiple tests using Benjamini-Hochberg correction [48]. Two-way ANOVA was used to determine if the expanded models of these GLMs significantly improved the response variable relative to the null model. Beta-diversity models were generated using methods described previously [44]. Briefly, we evaluated three beta-diversity metrics—Bray-Curtis, Canberra, and Sørensen and resolved the relationship between experimental parameters and beta-diversity by applying a step-wise model selection approach as implemented in the capscale function (vegan package v 2.5) [49]. Optimal models were subsequently subject to PERMANOVA analysis to determine if the selected model parameters significantly explained the variation in microbiome composition across samples. Differential abundance was measured using ANCOM-BC (v 2.0.1) [50].

### Electronic supplementary material

Below is the link to the electronic supplementary material.

Additional file 1: Contains supplementary statistical tables and figures from analyses.

Additional file 2: Proximate analysis of diets used in the study.

## Data Availability

All code generated during this analysis is available in the ZF Diet Infection 2020 repository at the following URL: https://github.com/sielerjm/ZF-Diet_Infection. The raw sequence files generated during the current study are available at the NCBI Sequence Read Archive (SRA) project numbers PRJNA929305.

## List of Abbreviations

dpf: Days post fertilization
BCS: Body condition score
ZIRC: Zebrafish International Resource Center
