# Supplementary material for: Disentangling the link between zebrafish diet, gut microbiome succession, and Mycobacterium chelonae infection
Source: Anim Microbiome. 2023 Aug 10;5:38. doi: 10.1186/s42523-023-00254-8 (PMC10413624; doi:10.1186/s42523-023-00254-8)
Supplement: Supplementary file 1 — Additional file 1: Contains supplementary statistical tables and figures from analyses. [file 42523_2023_254_MOESM1_ESM.docx]

**Supplementary Tables and Figures**

1. **Diet**

**1.1) Body Size**

**1.1.2)**

**
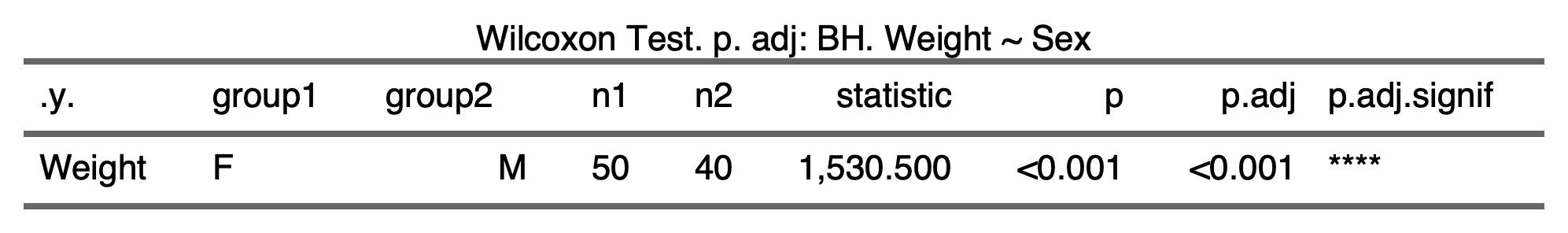
**

**1.1.3)**

**
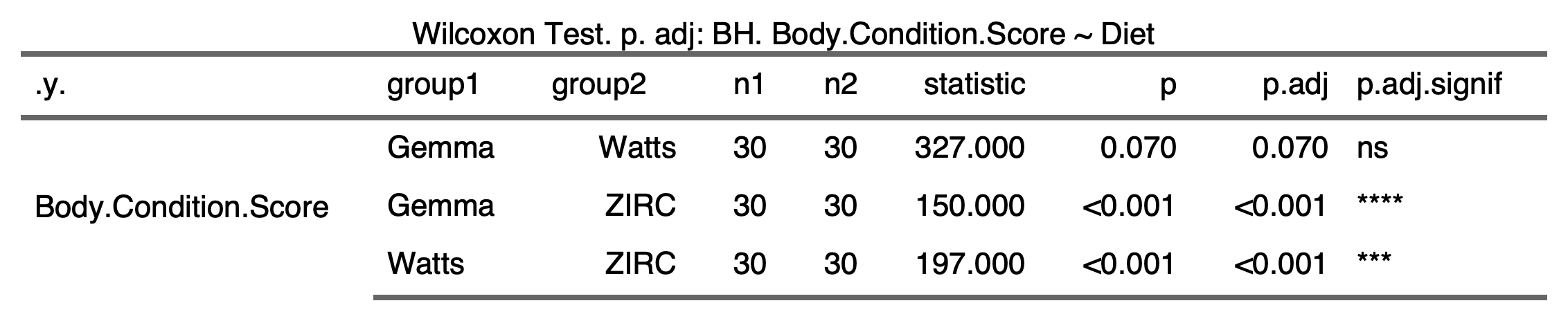
**

**1.1.4)**

**
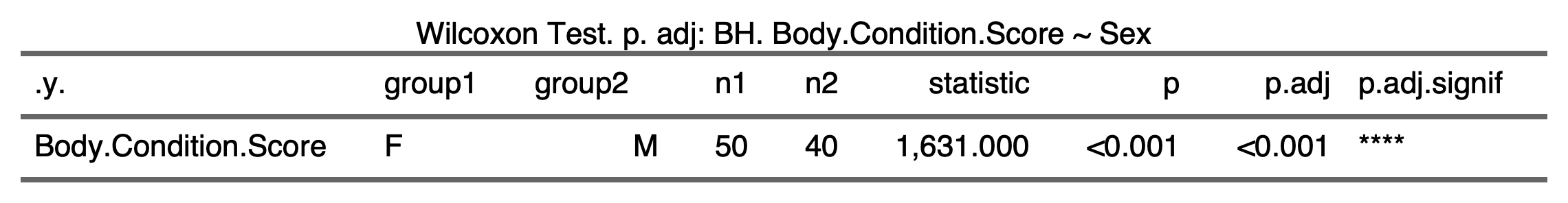
**

**1.2) Alpha Diversity**

**1.2.1)**

**
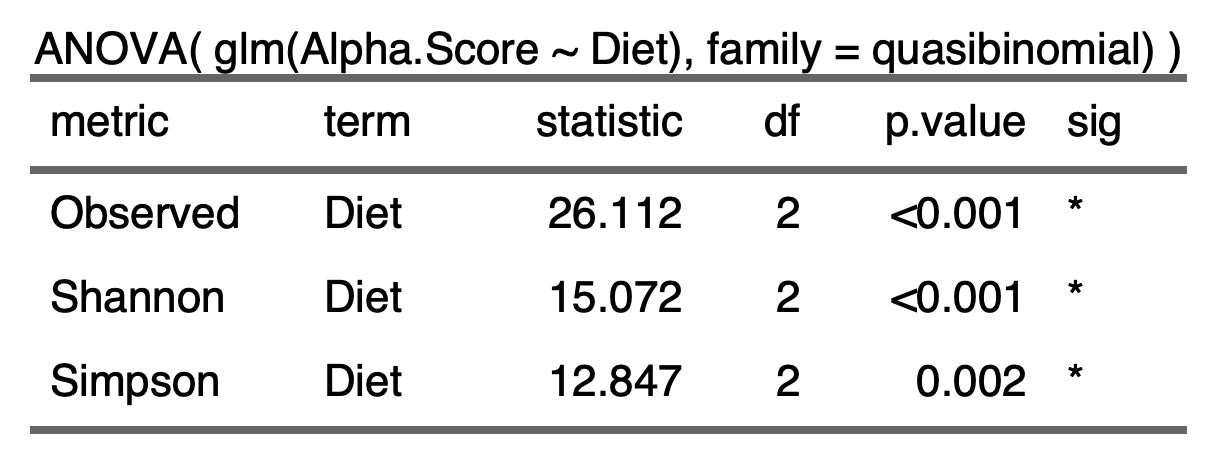
**

**1.2.2)**

**
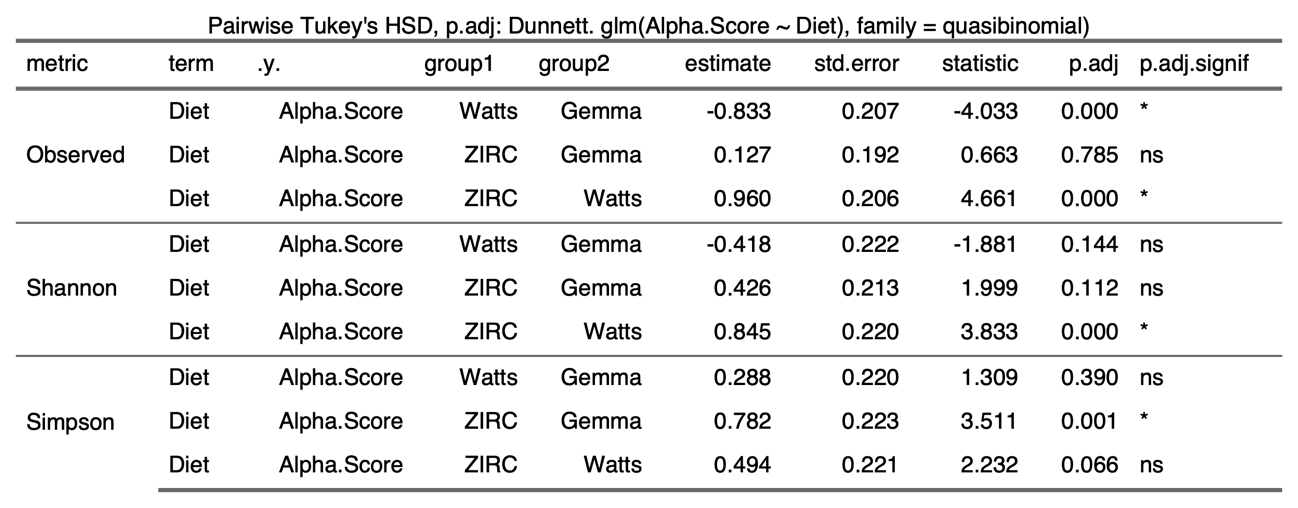
**

**1.3) Beta-diversity**

**1.3.1)**

**
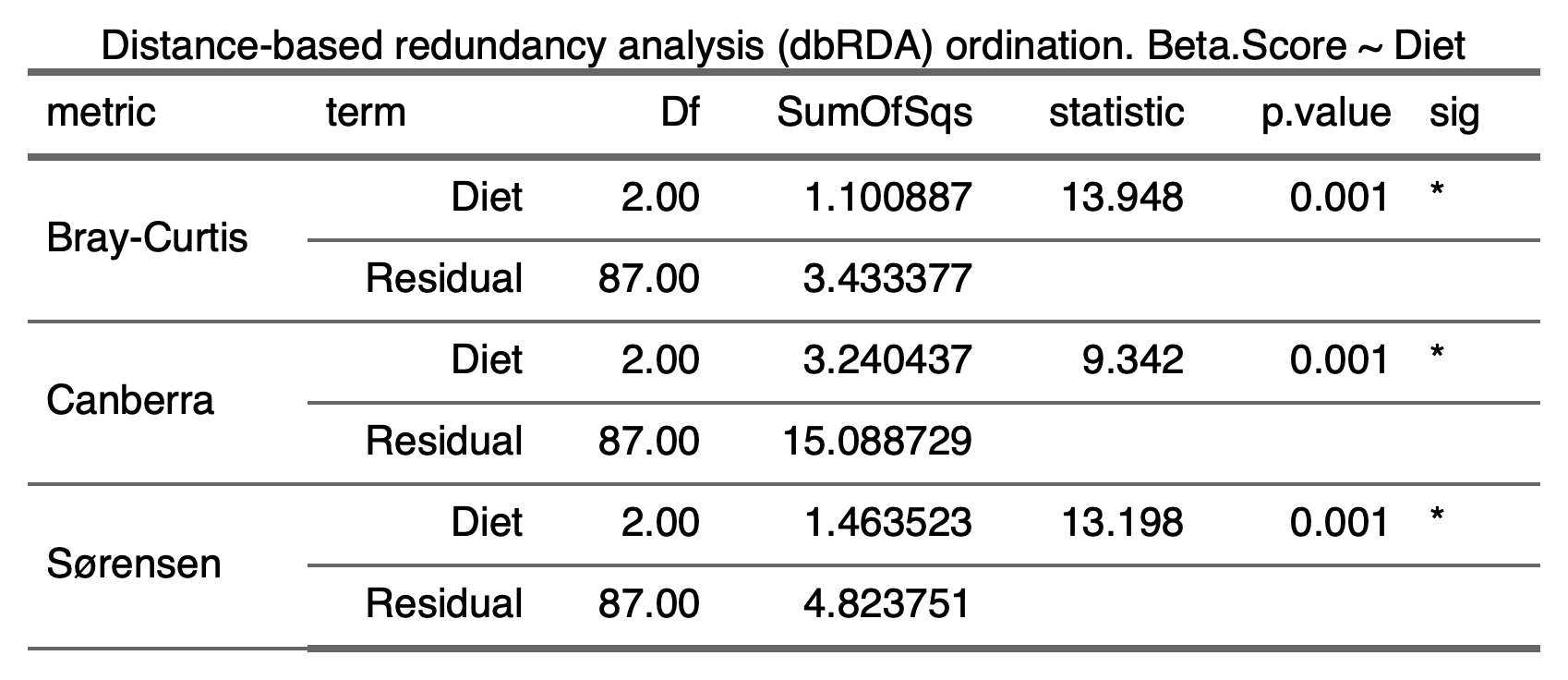
**

**1.4) Beta-Dispersion**

**1.4.1)**

| **Bray-Curtis** | **Canberra** | **Sørensen** |
| --- | --- | --- |
| **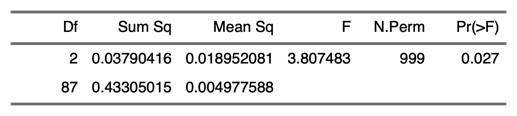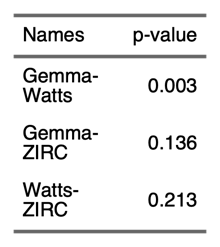** | **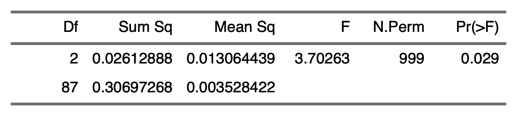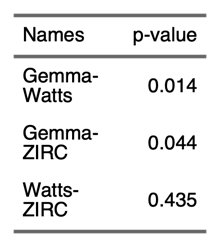** | **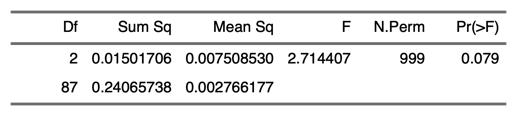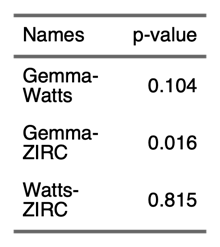** |

**1.5) Differential Abundance**

**1.5.1)**

**
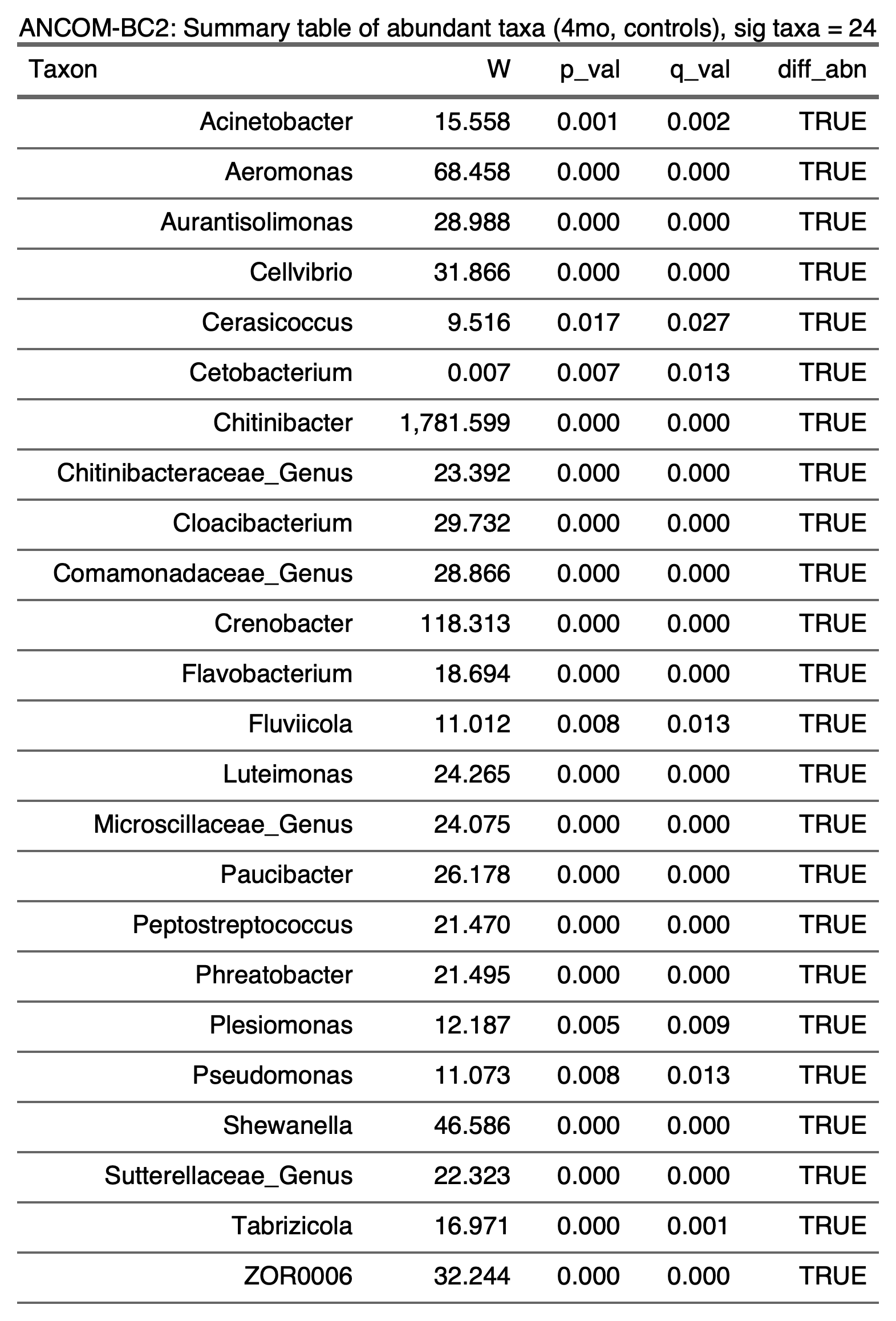
**

1. **Diet and development**

**2.1) Body Size**

**2.1.1)**

**
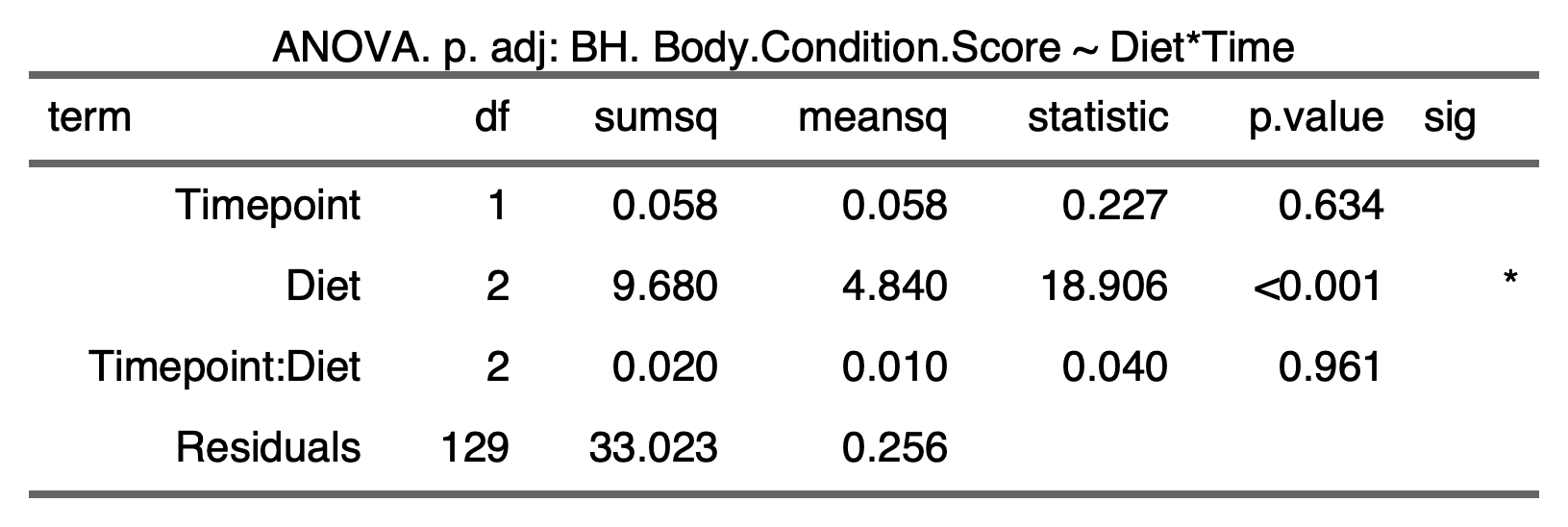
**

**2.1.2) Microbiome**

**2.1.2.1) Alpha Diversity (214 dpf)
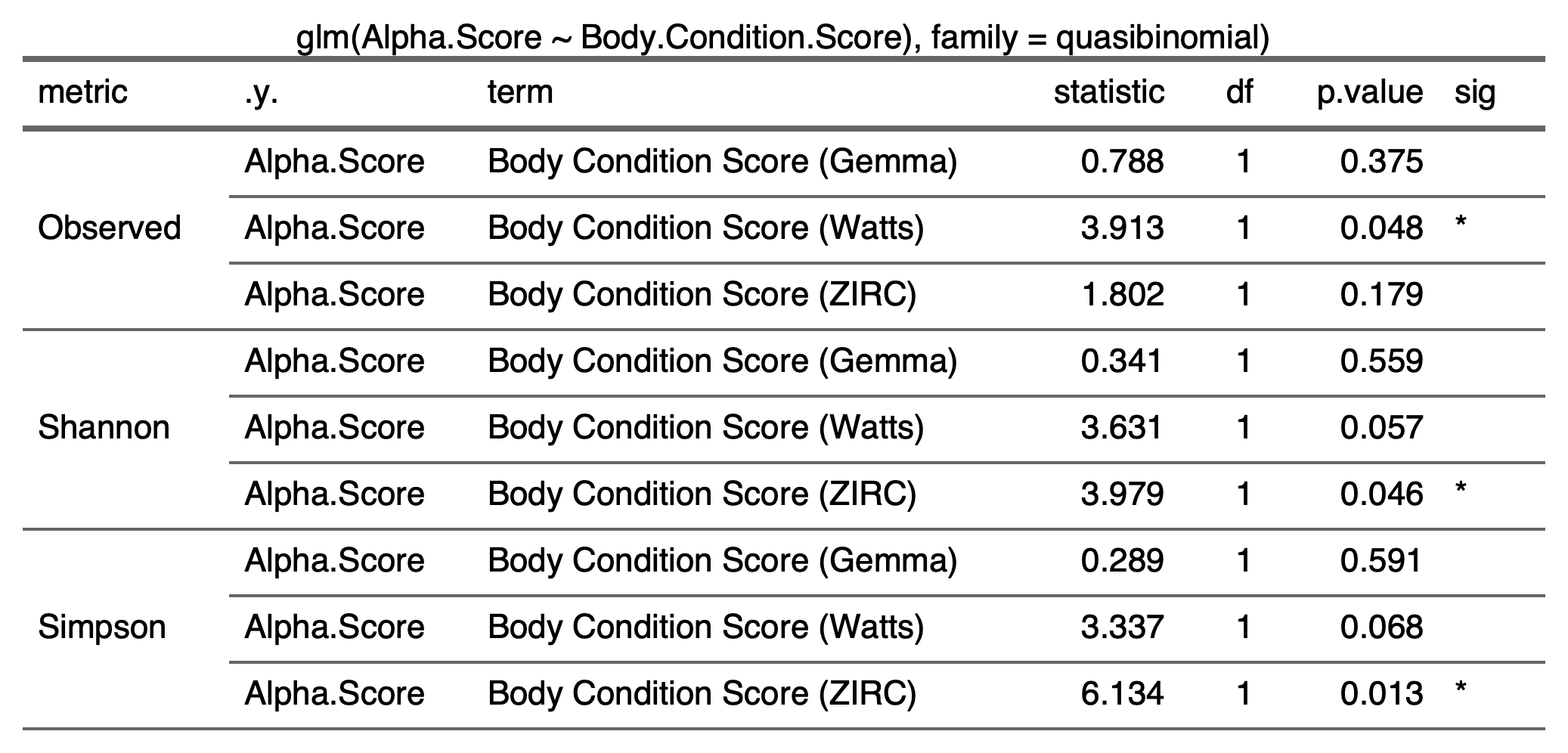
**

**2.1.2.2) Beta Diversity**

**
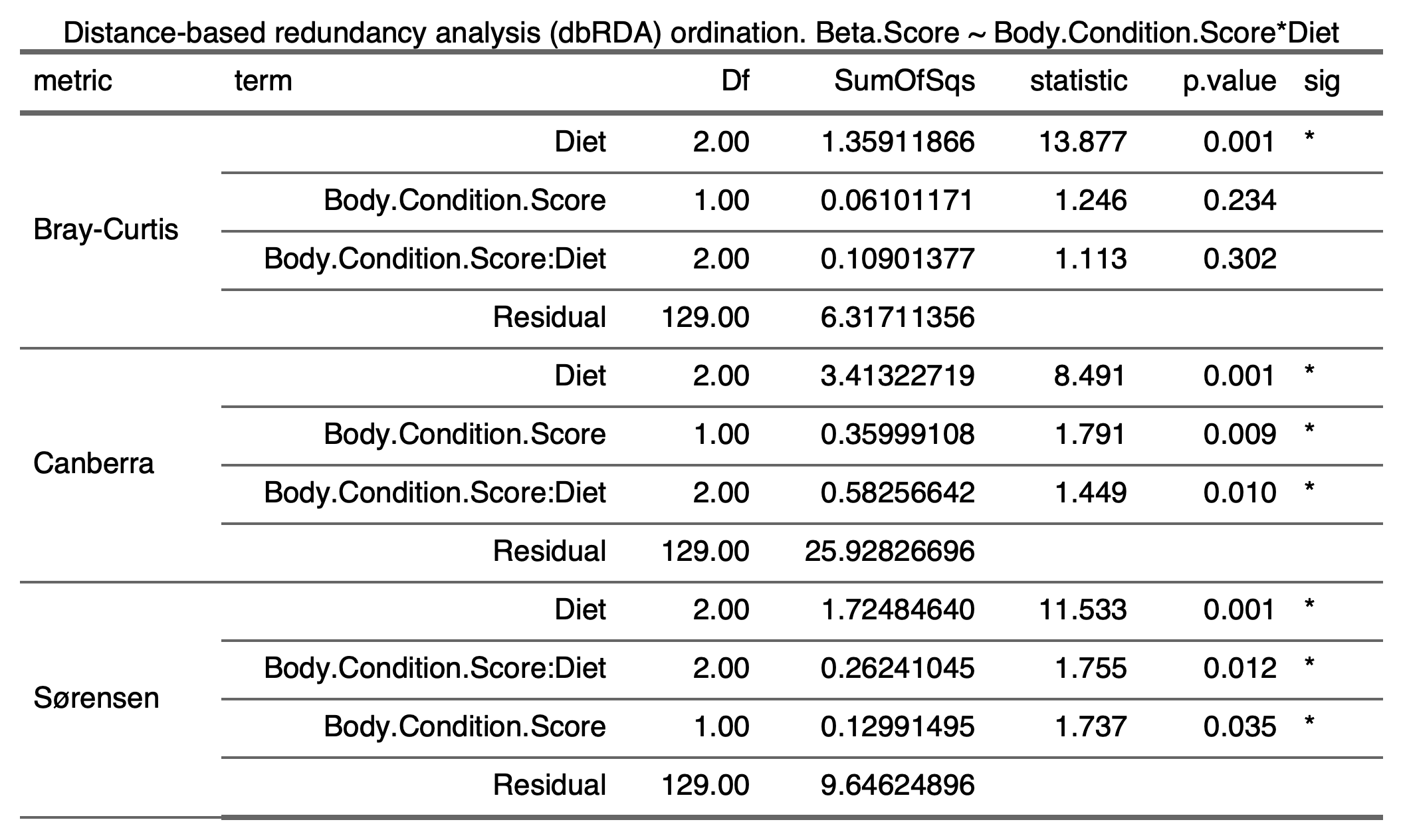
**

**2.1.3) 214 dpf**

**2.1.3.1) Body Size**

**
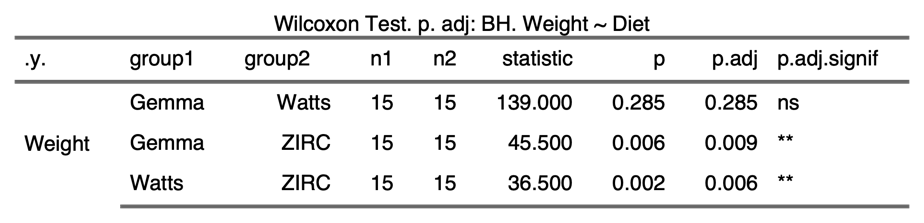
**

**2.1.3.2) Alpha Diversity**

**
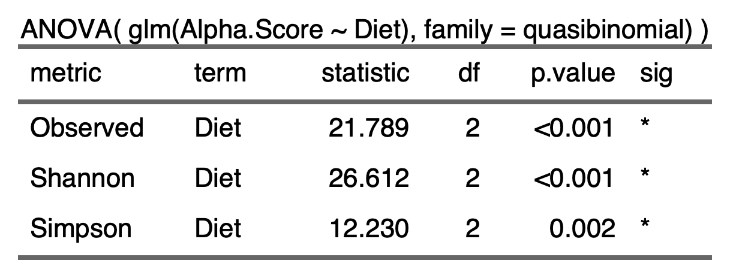
**

**2.1.3.3) Beta Diversity**

**
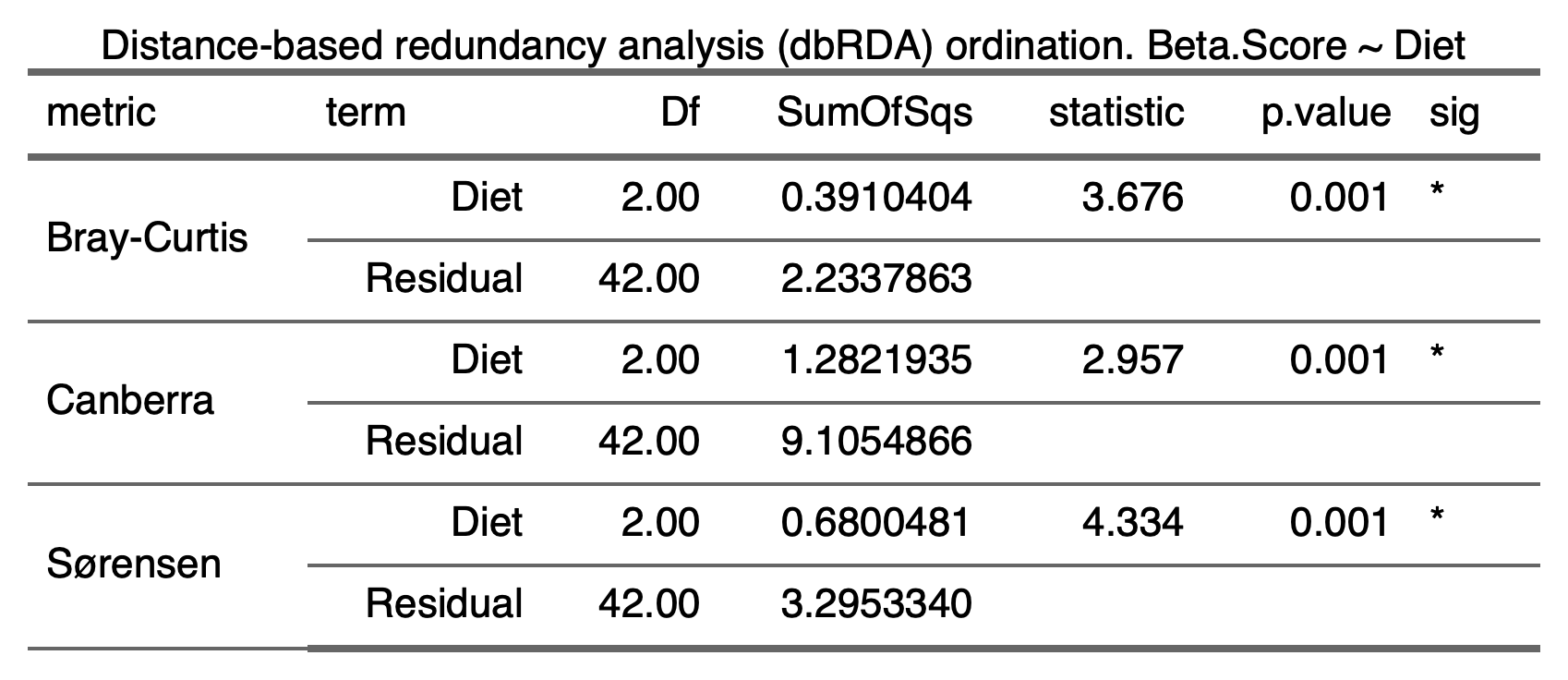
**

**2.1.3.4) Beta diversity**

| **Bray-Curtis** | **Canberra** | **Sørensen** |
| --- | --- | --- |
| **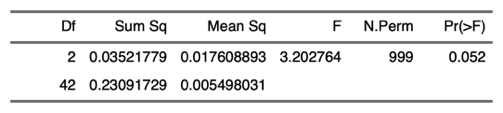**  **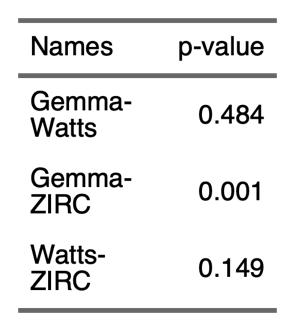** | **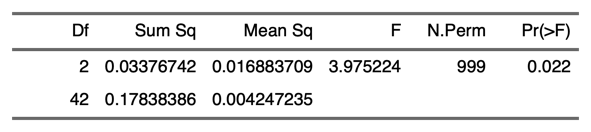**  **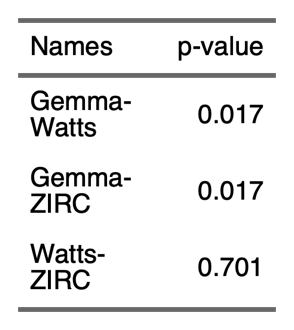** | **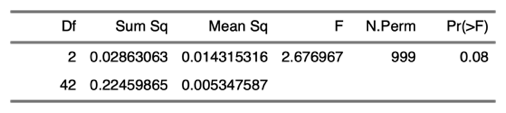**  **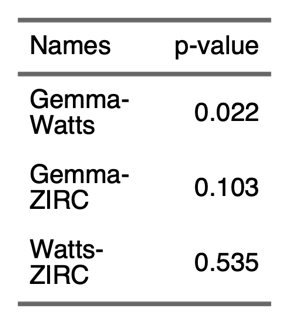** |

**2.2) Alpha Diversity**

**2.2.1)**

**
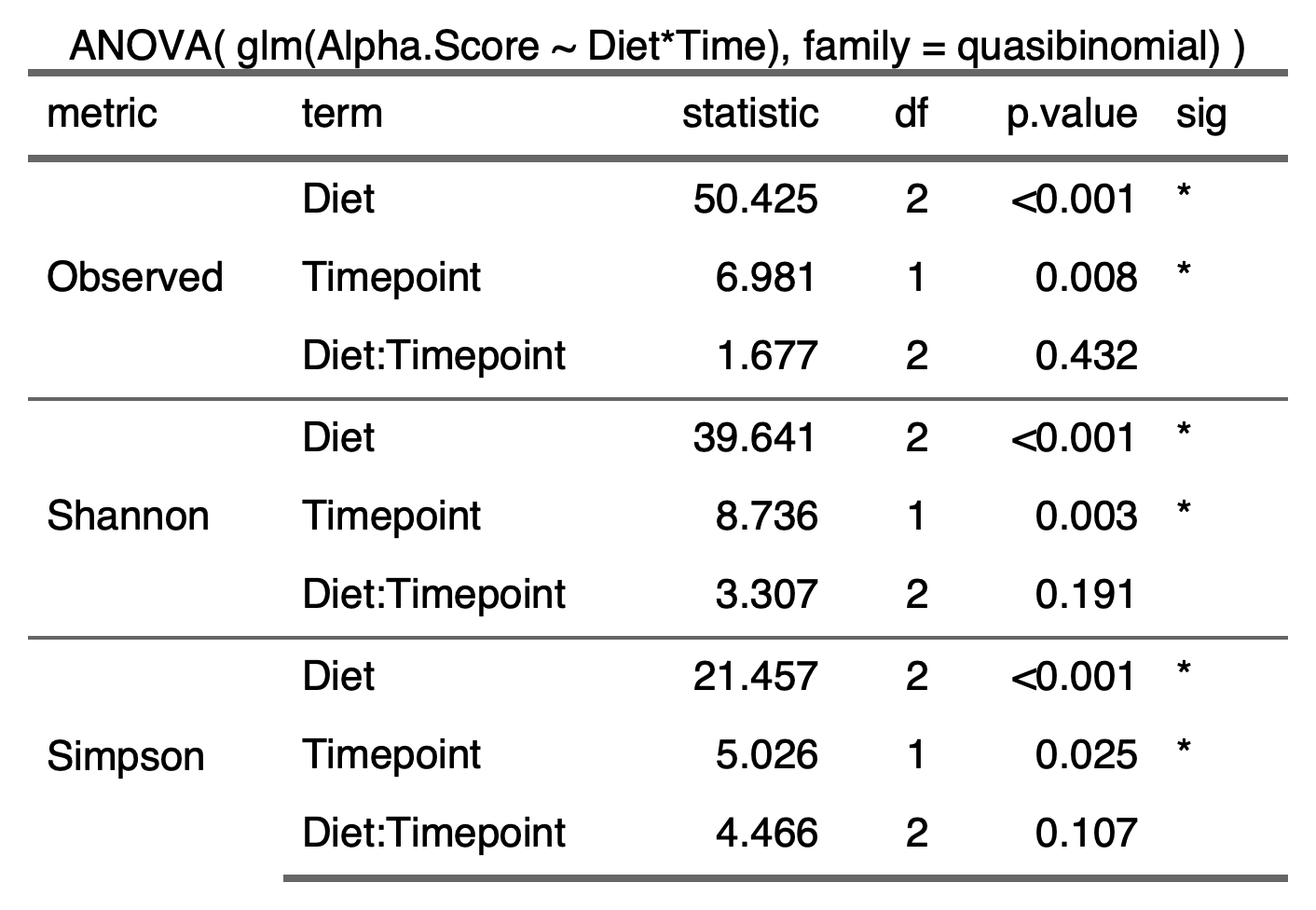
**

**2.2.2)**

**
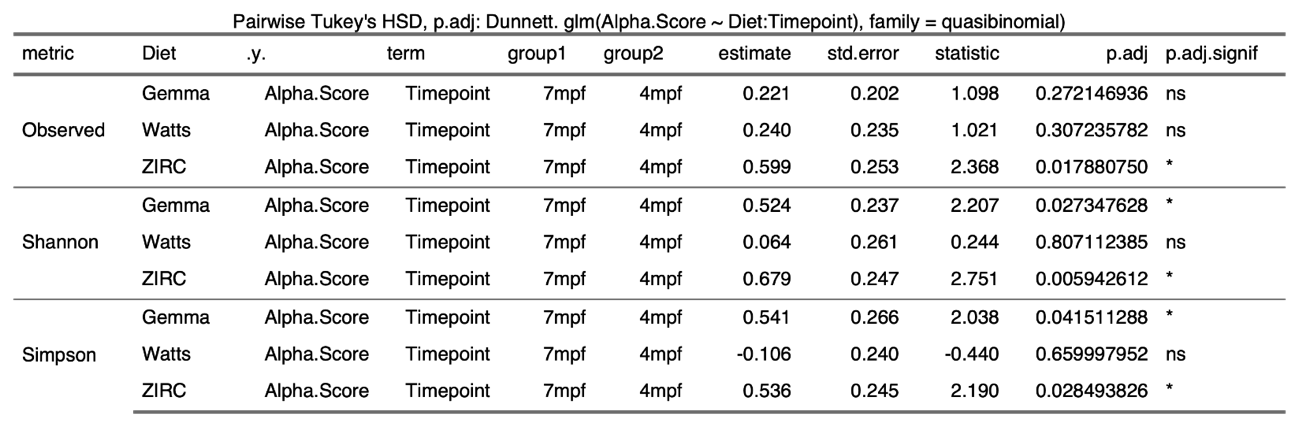
**

**2.3) Beta Diversity**

**2.3.1)**

**
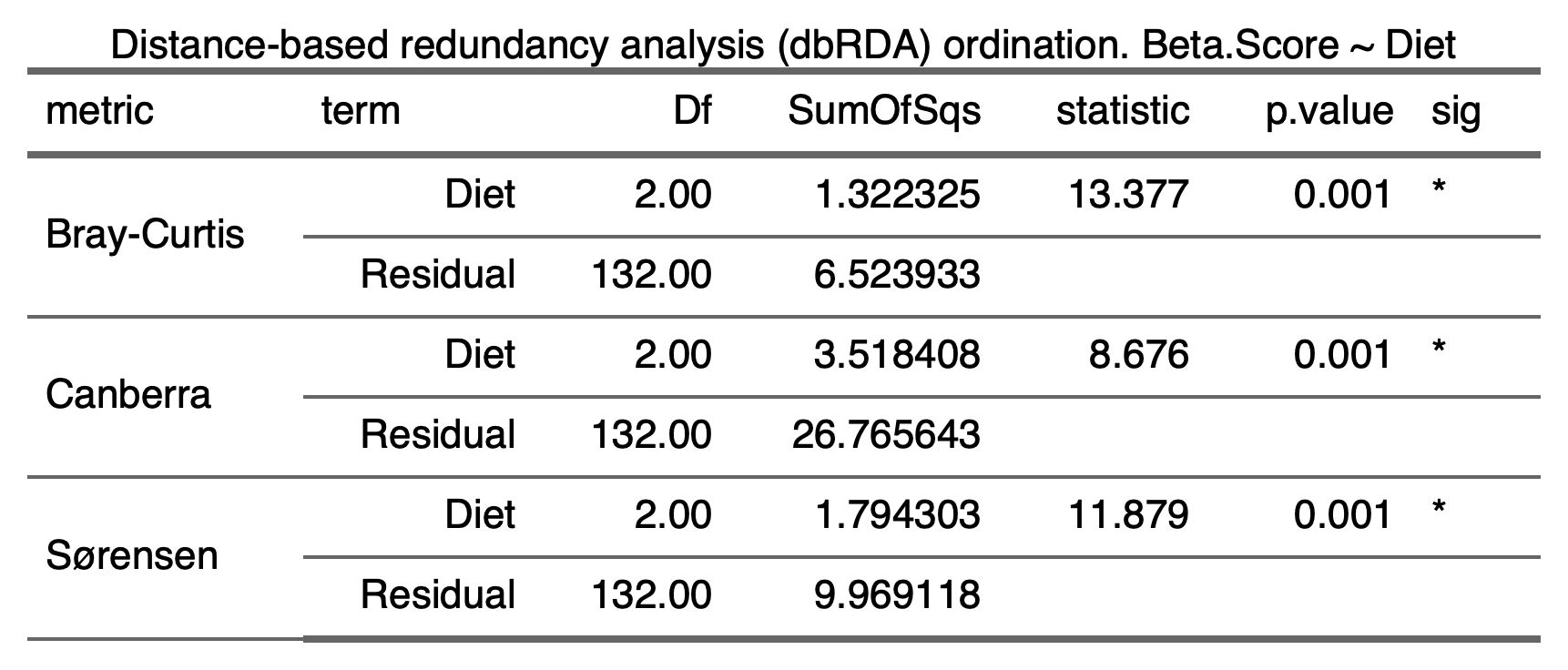
**

**2.3.2)**

**
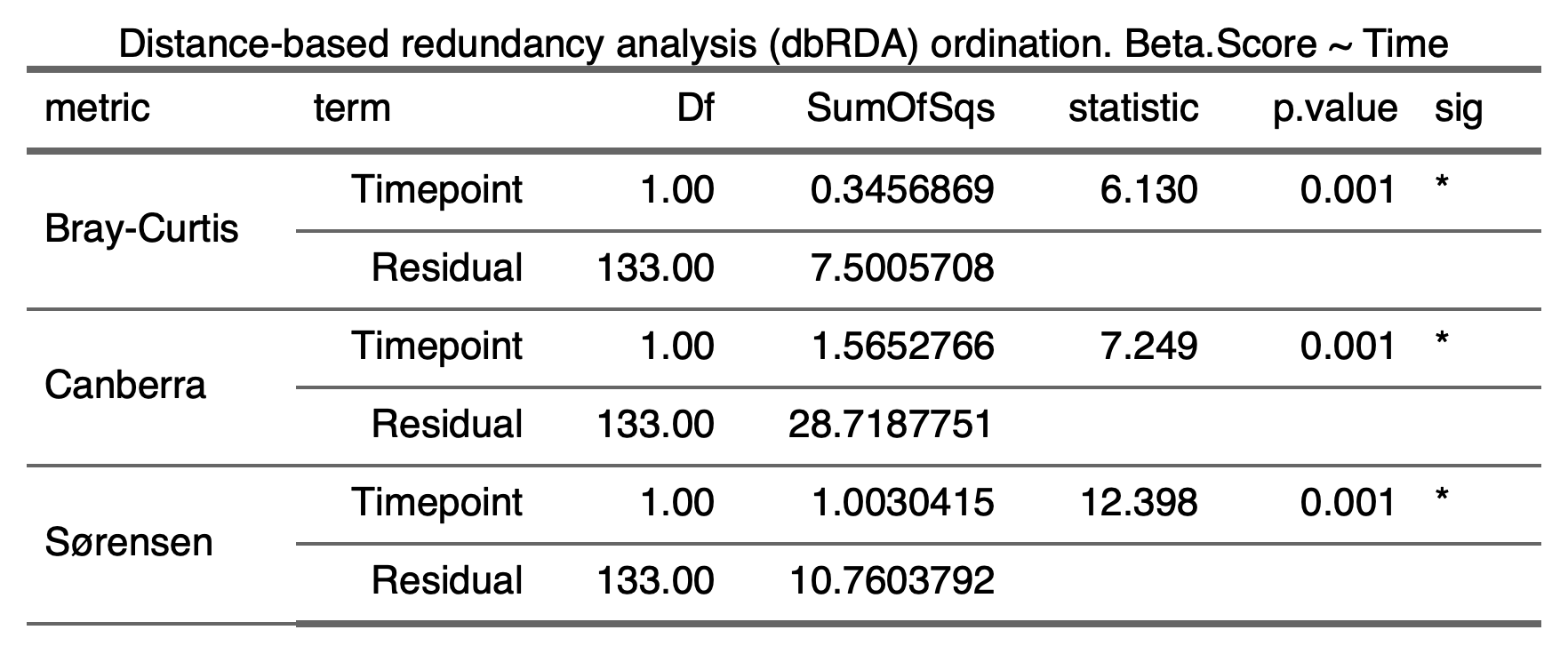
**

**2.4) Beta Dispersion**

**2.4.1) Diet**

**2.4.1.1) Gemma Diet**

| **Bray-Curtis** | **Canberra** | **Sørensen** |
| --- | --- | --- |
| **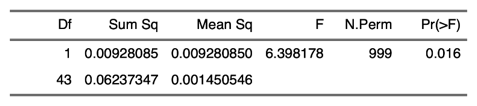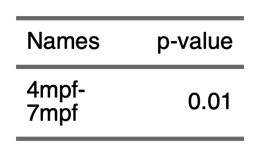** | **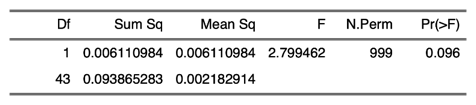**  **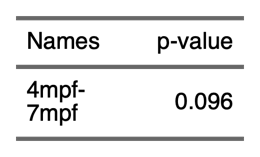** | **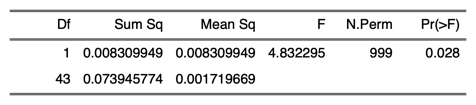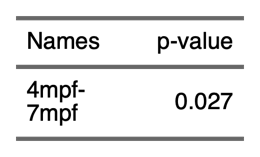** |

**2.4.1.2) Watts Diet**

| **Bray-Curtis** | **Canberra** | **Sørensen** |
| --- | --- | --- |
| **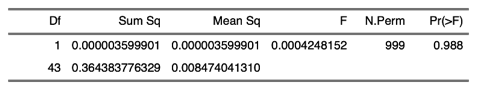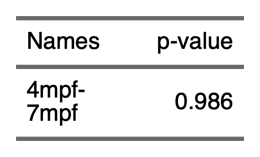** | **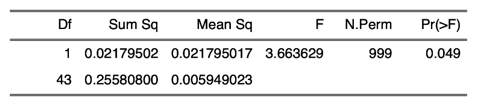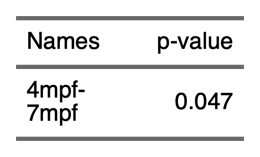** | **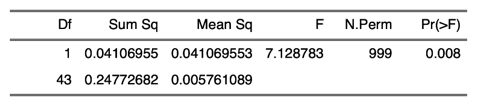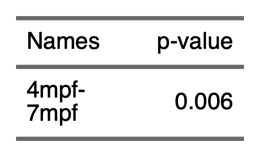** |

**2.4.1.3) ZIRC Diet**

| **Bray-Curtis** | **Canberra** | **Sørensen** |
| --- | --- | --- |
| **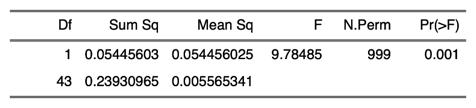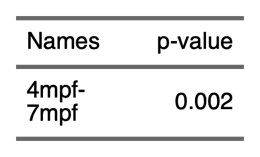** | **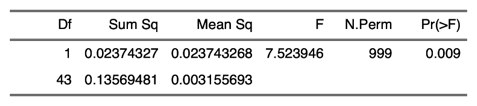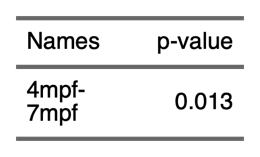** | **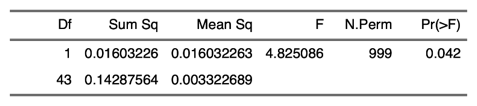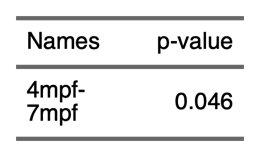** |

**2.5) Differential Abundance**

**2.5.1) All diets (129 vs 214 dpf)**

**
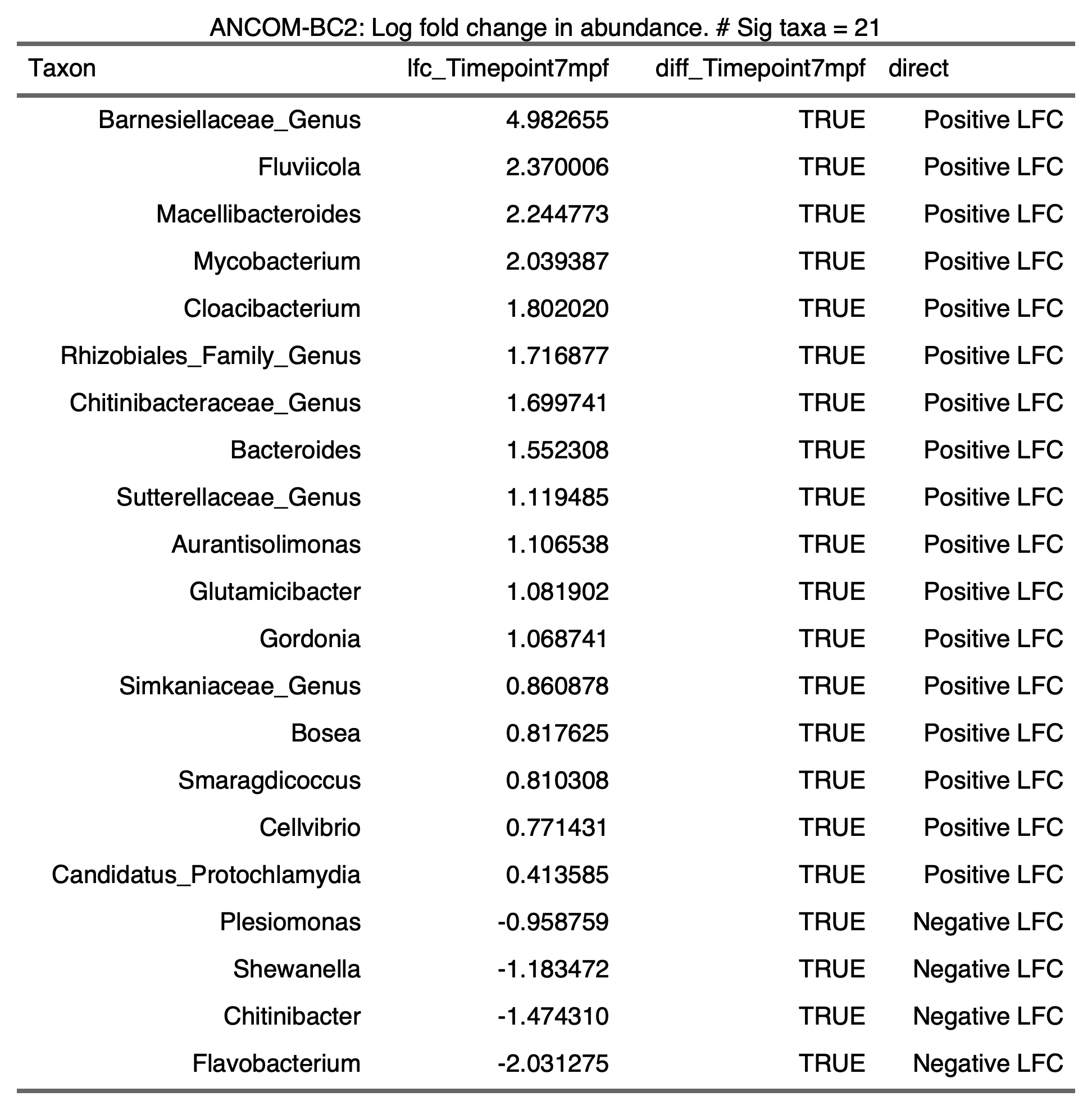
**

**2.5.2) Gemma Diet**

**
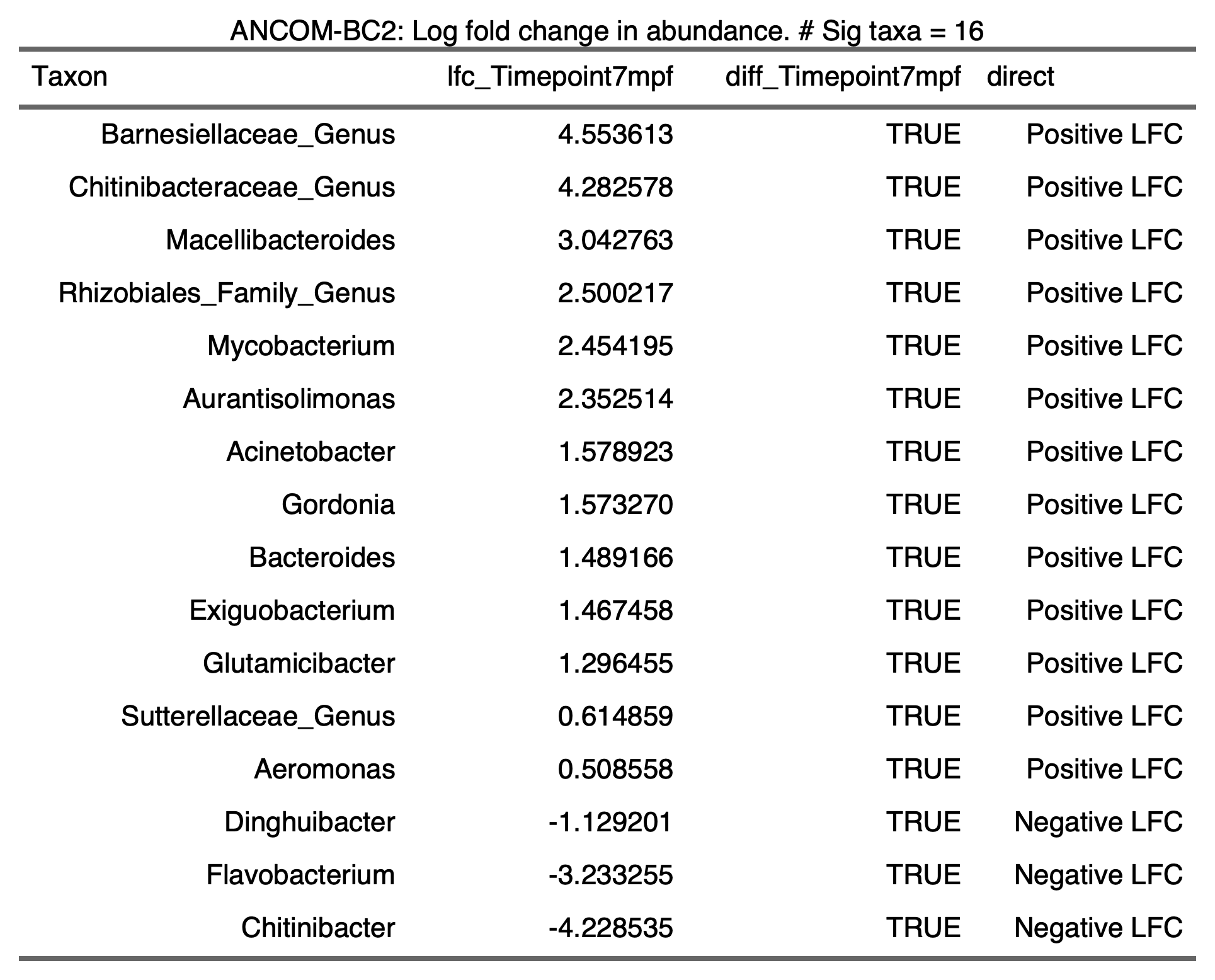
**

**2.5.3) Watts Diet**

**
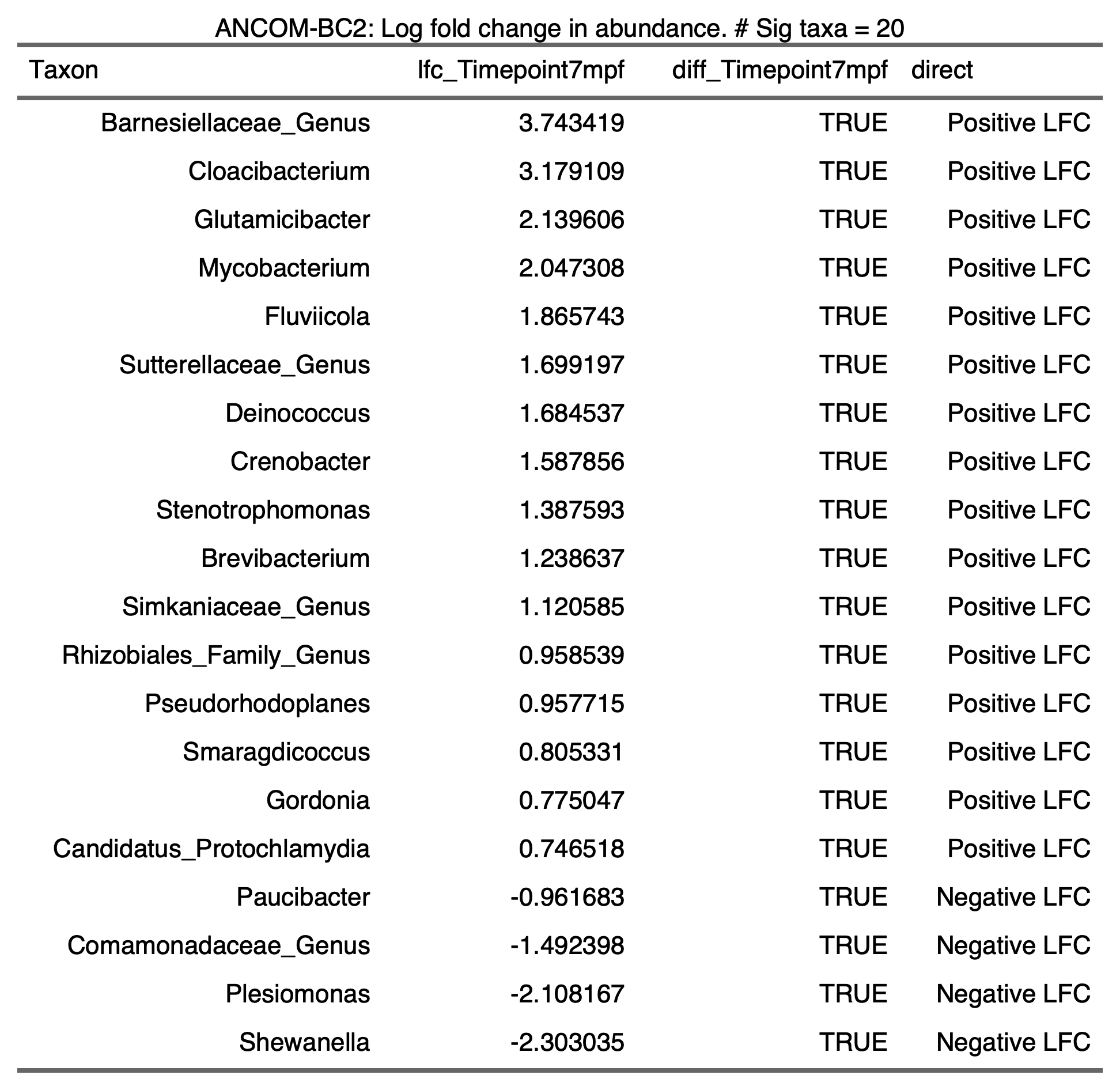
**

**2.5.4) ZIRC Diet**

**
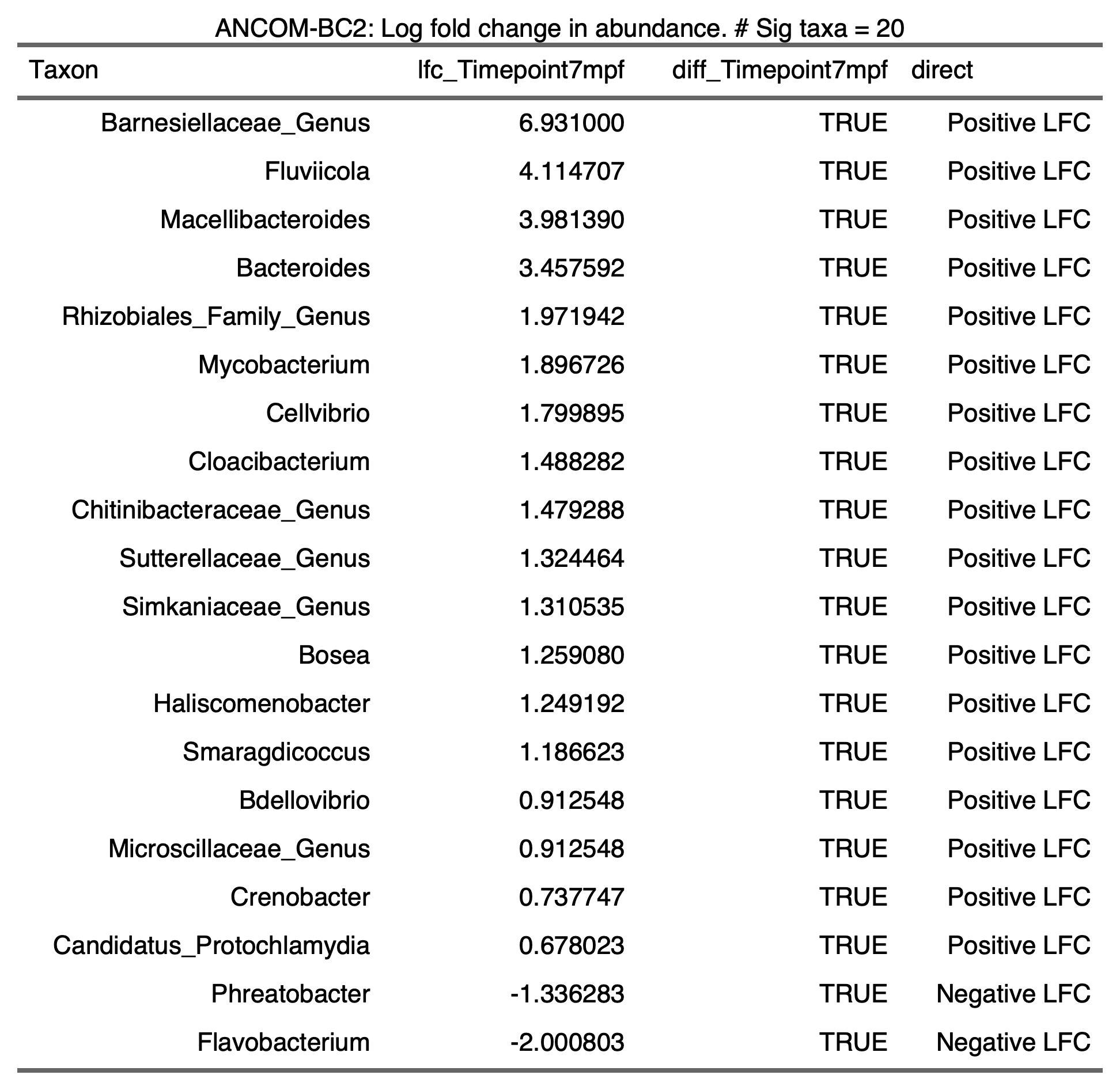
**

1. **Pathogen Exposure**

**3.1) Histopathology**

**3.1.1) Infection Counts ~ Diet + Sex**

**
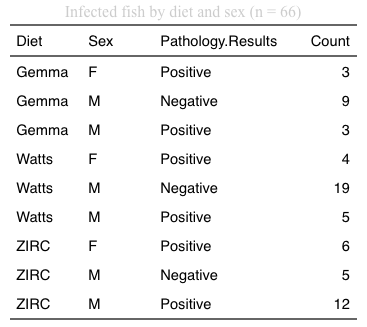
**

**Asymptotic Pearson Chi-Squared Test**

**data: Var2 by Var1 (p, n)**

**chi-squared = 11.519, df = 2, p-value = 0.003152**

**3.1.2) Infection Counts ~ Diet (Males only)**

**
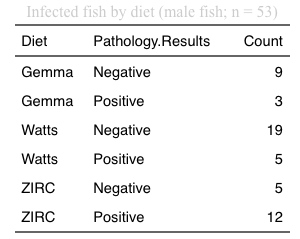
**

**Asymptotic Pearson Chi-Squared Test**

**data: Var2 by Var1 (p, n)**

**chi-squared = 11.556, df = 2, p-value = 0.003096**

**3.1.3.1) Infection Counts ~ Diet (Males and Females), microbiome sample subset**


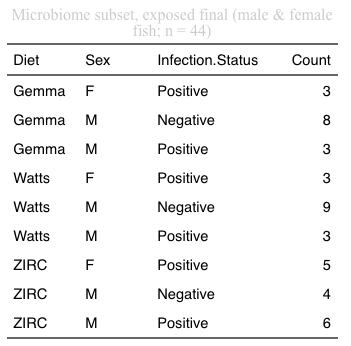


**Asymptotic Pearson Chi-Squared Test**

**data: Var2 by Var1 (p, n)**

**chi-squared = 4.0699, df = 2, p-value = 0.1307**

**3.1.3.2) Infection Counts ~ Diet (Males Only), microbiome sample subset**

**
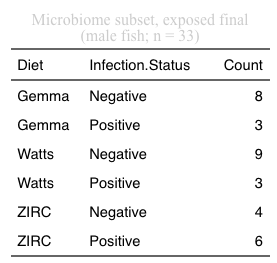
**

**Asymptotic Pearson Chi-Squared Test**

**data: Var2 by Var1 (p, n)**

**chi-squared = 3.4768, df = 2, p-value = 0.1758**

**3.1.4.1) Body Condition Score ~ Diet*Infection Counts**

**
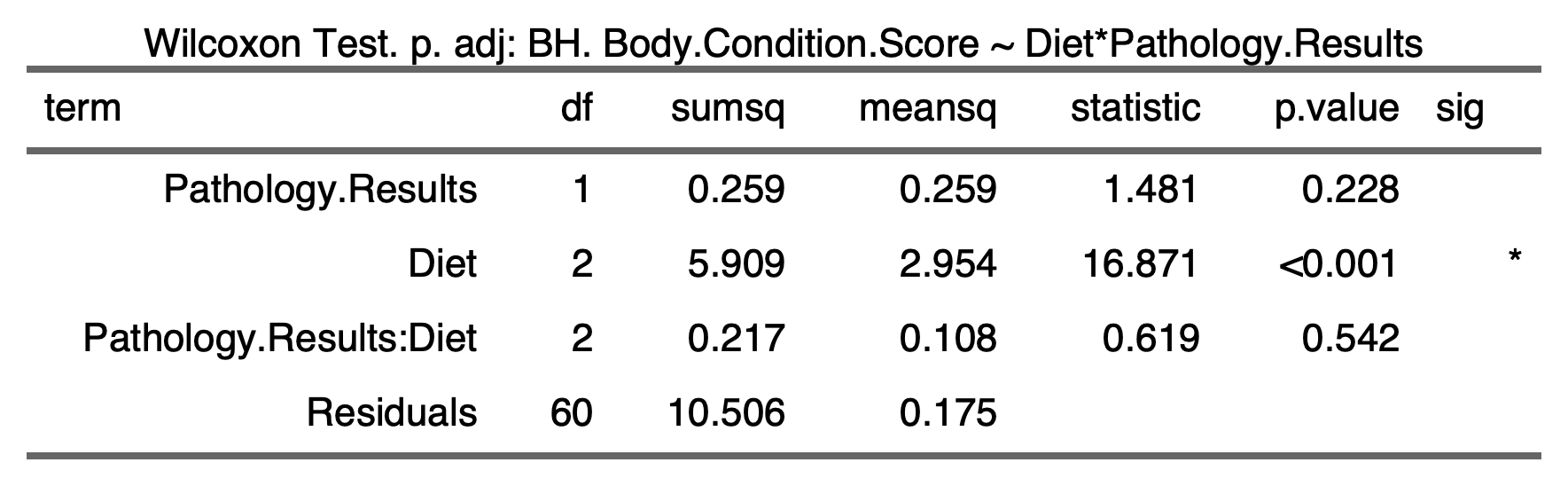
**

**3.1.4.2) Male Fish: Body Condition Score ~ Diet*Infection Counts**

**
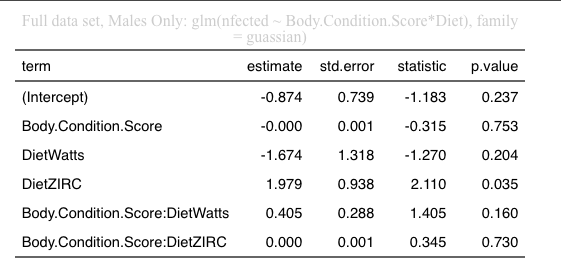
**

**3.1.5) Alpha Diversity**

**
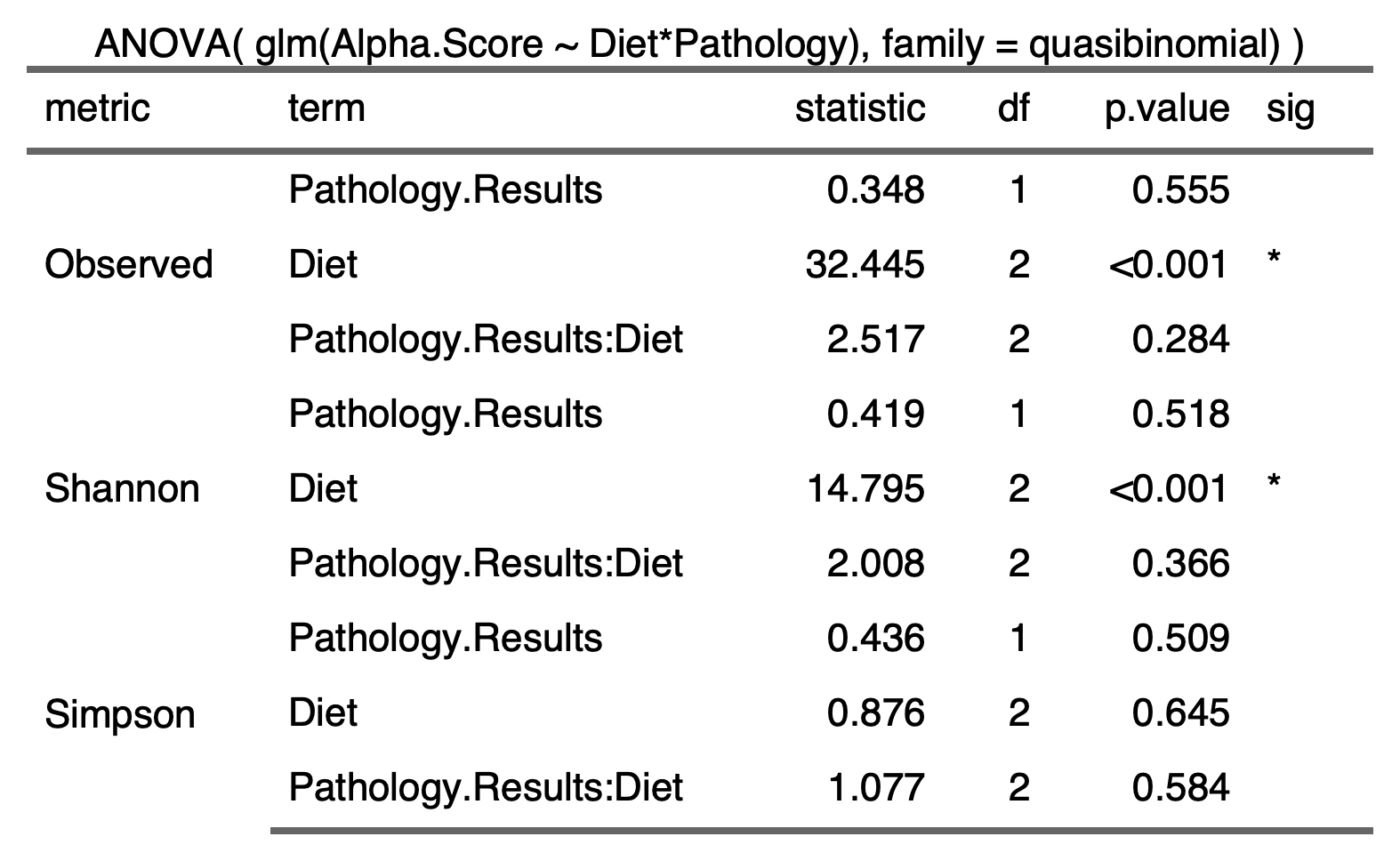
**

**3.1.6) Beta Diversity**

**
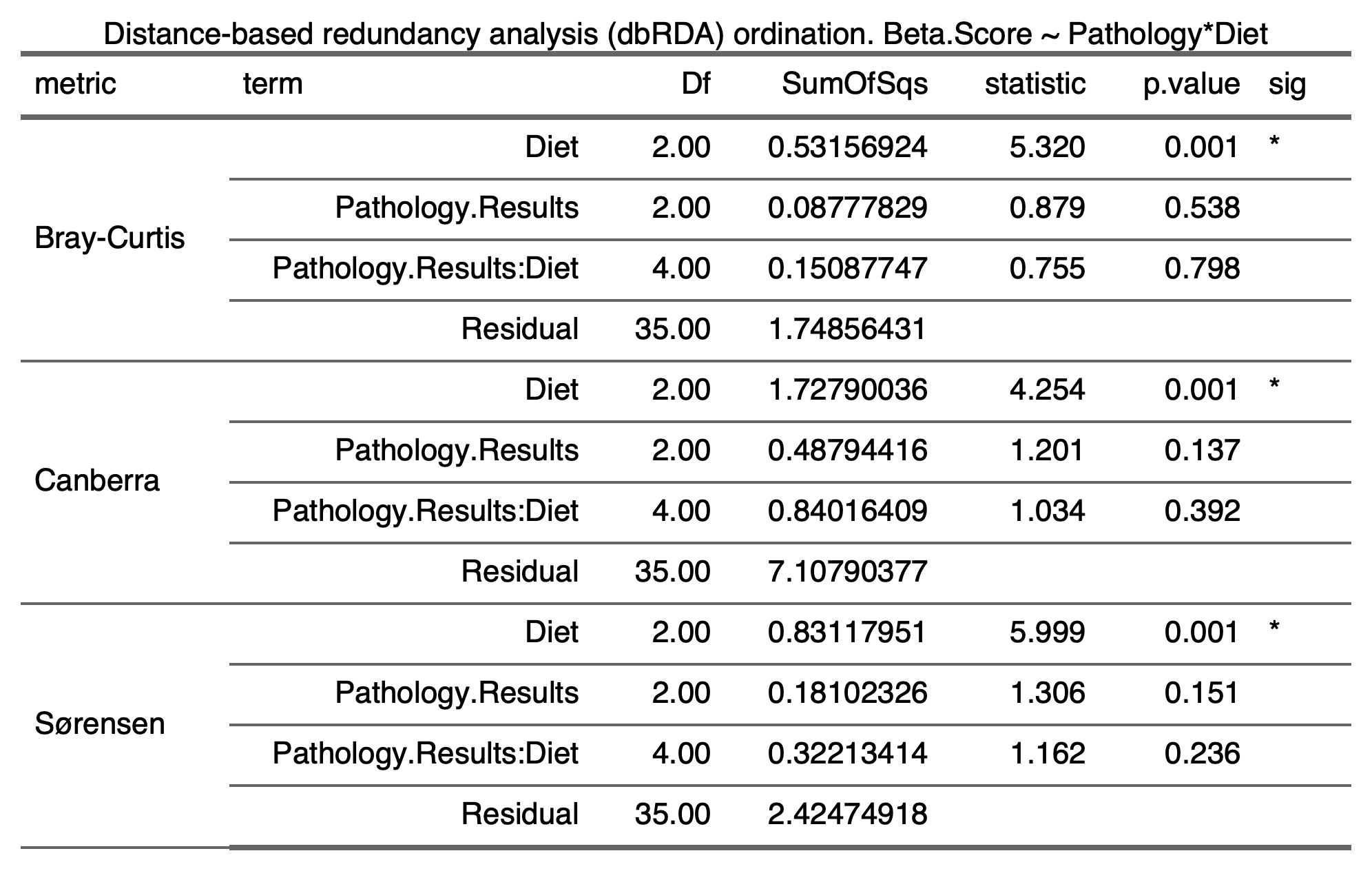
**

**3.1.7) Infection Counts ~ Dose:Timepoint**

**Compare infection counts between Mycobacterium doses of raw data set of unsubsetted fish (i.e., fish that were exposed to Mycobacterium chelonae were checked at 5 weeks post exposure (wpe) and 15 wpe for infection counts).**

**5 wpe fish:**

**
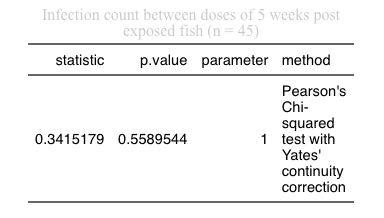
**

**15 wpe fish:**

**
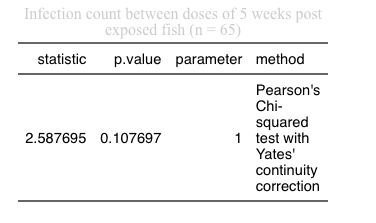
**

**Microbiome subset (infection counts between doses of fish used for microbiome analysis):**

**
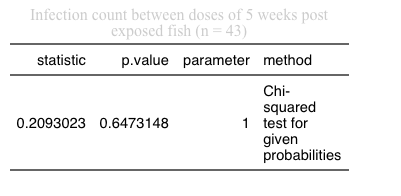
**

**3.2) Alpha Diversity**

**3.2.1)**

**
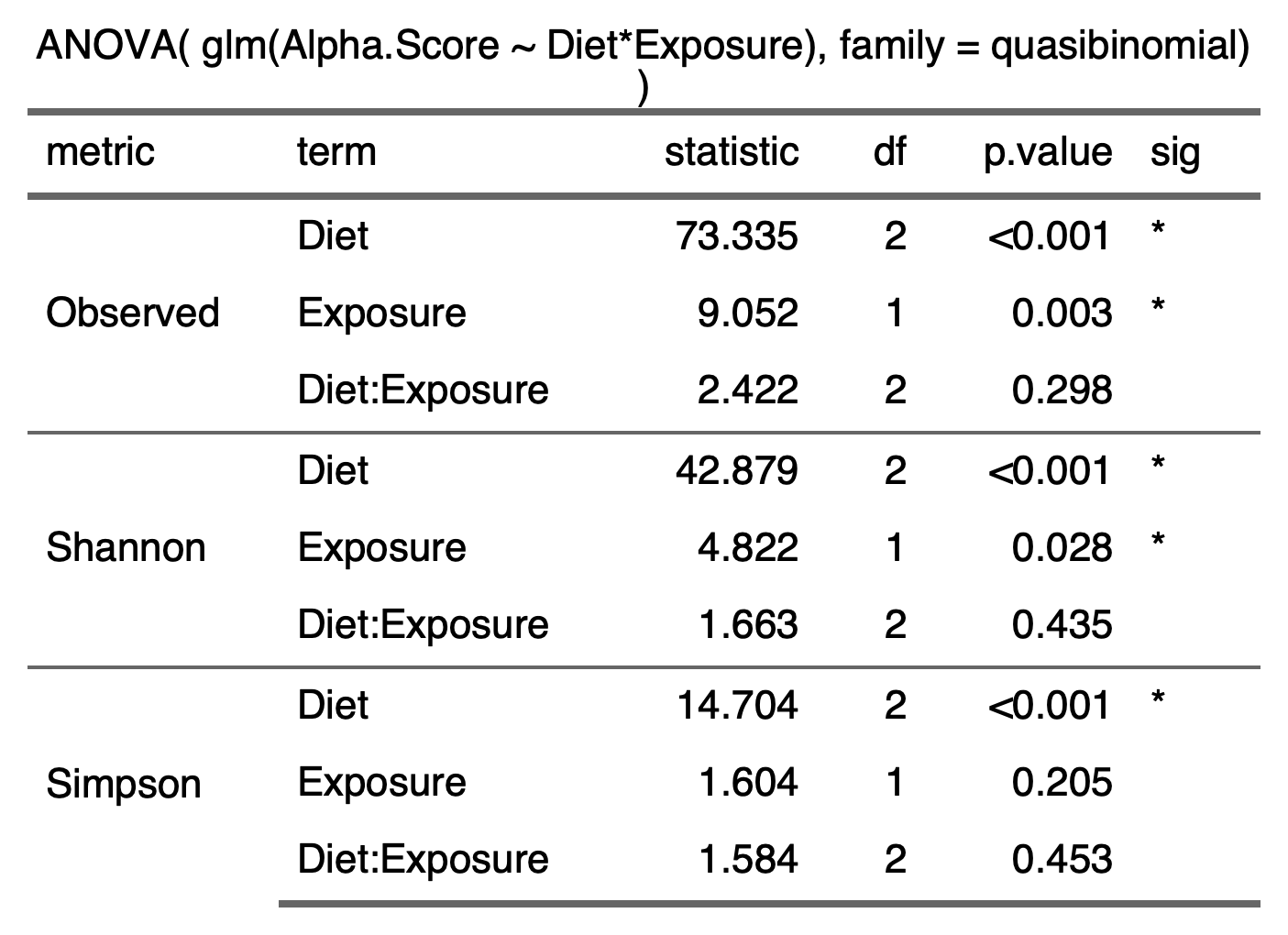
**

**3.2.2)**

**
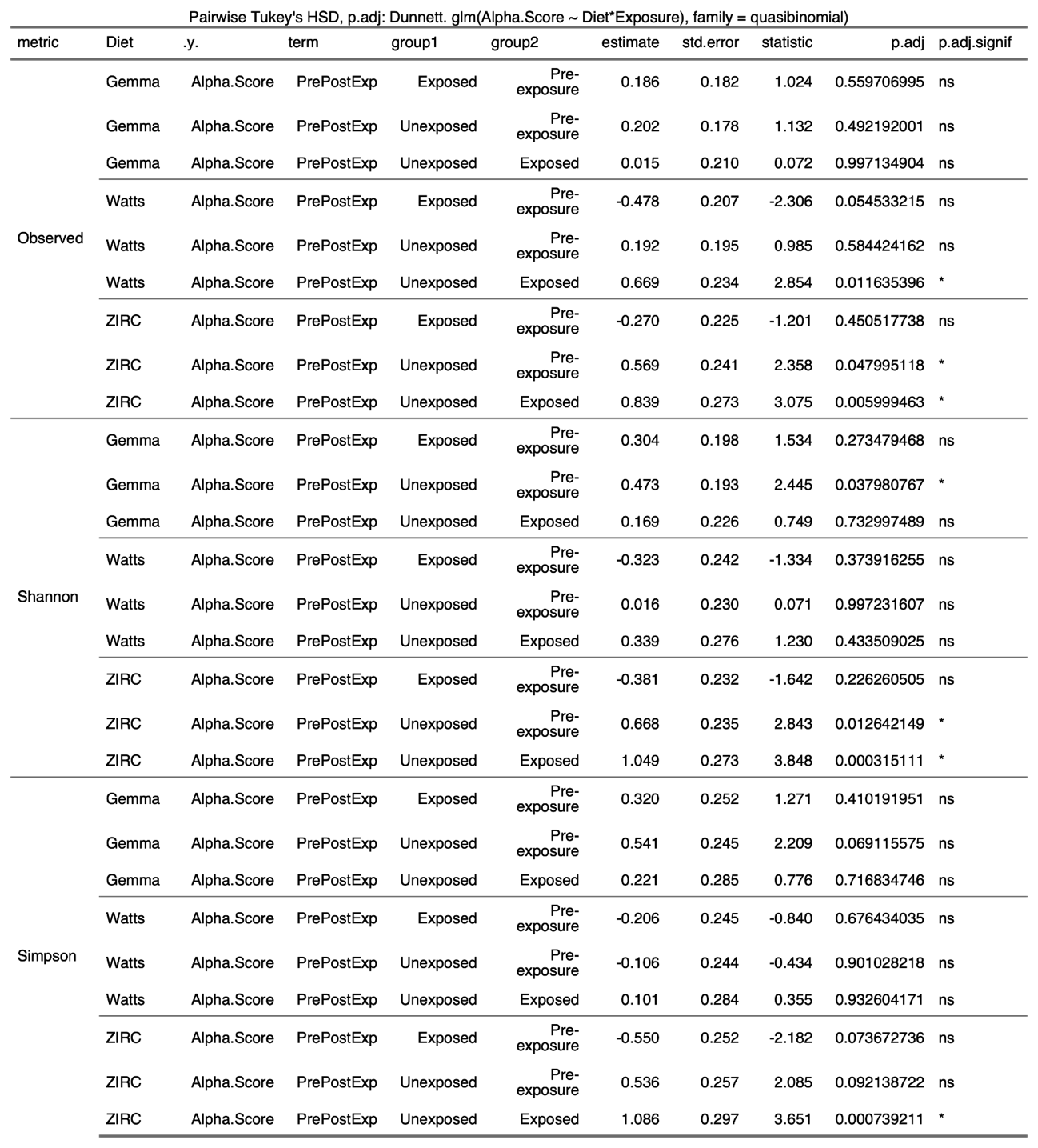
**

**3.3) Beta Diversity**

**3.3.1)**

**
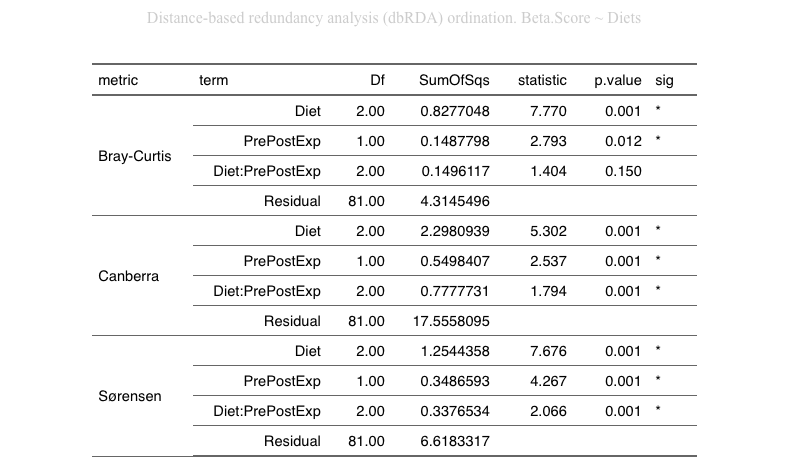
**

**3.4) Beta Dispersion**

**3.4.1) Gemma Diet**

| **Bray-Curtis** | **Canberra** | **Sørensen** |
| --- | --- | --- |
| **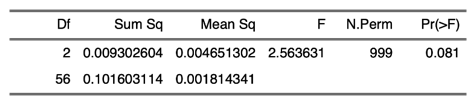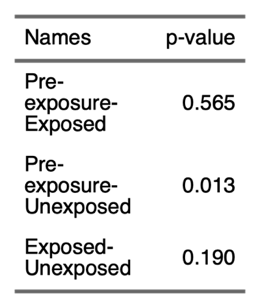** | **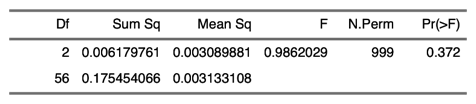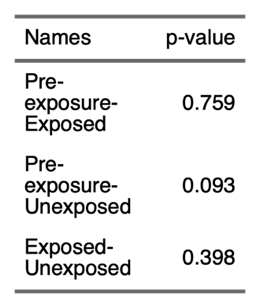** | **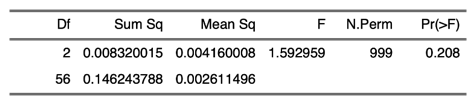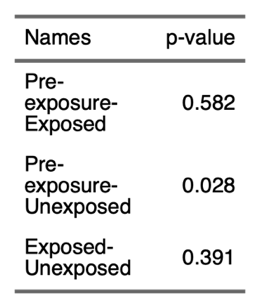** |

**3.4.2) Watts Diet**

| **Bray-Curtis** | **Canberra** | **Sørensen** |
| --- | --- | --- |
| **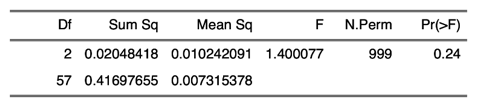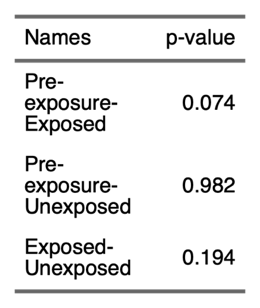** | **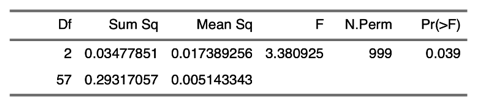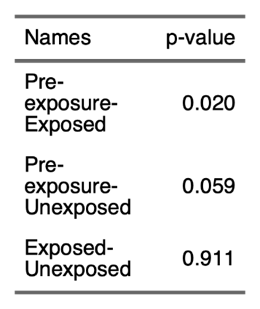** | **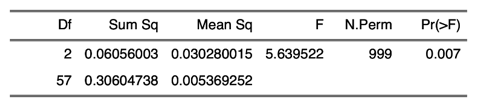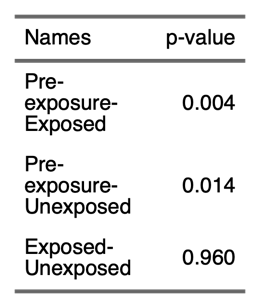** |

**3.4.3) ZIRC Diet**

| **Bray-Curtis** | **Canberra** | **Sørensen** |
| --- | --- | --- |
| **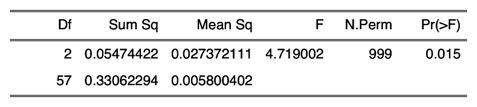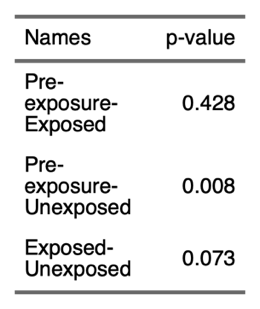** | **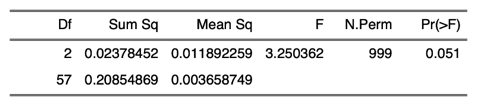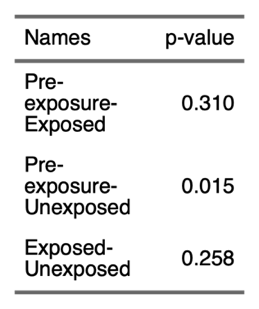** | **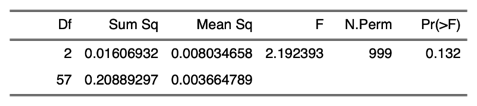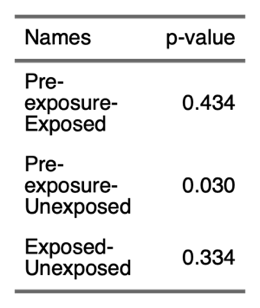** |

**3.5) Differential Abundance**

**3.5.1) All Diets**

**
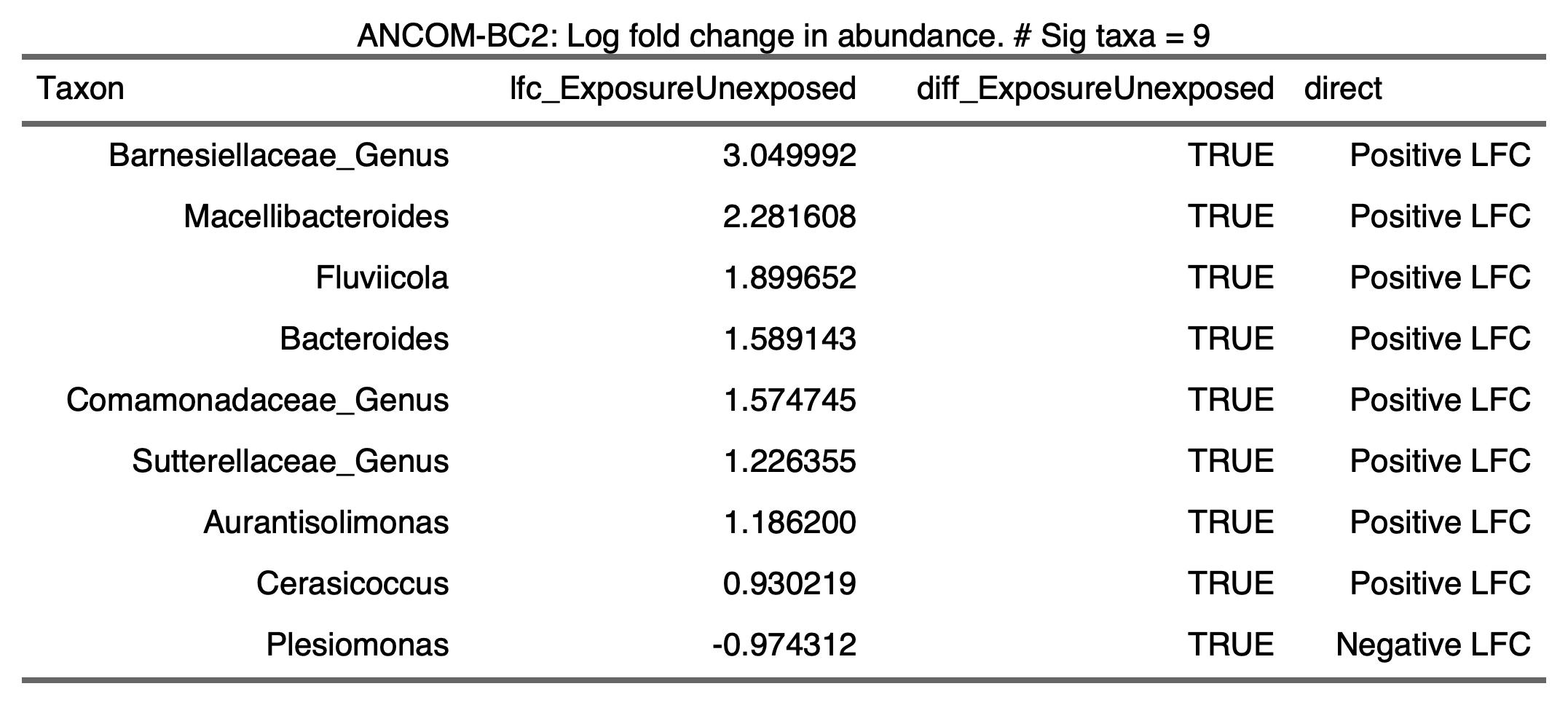
**

**3.5.2) Gemma Diet**

**
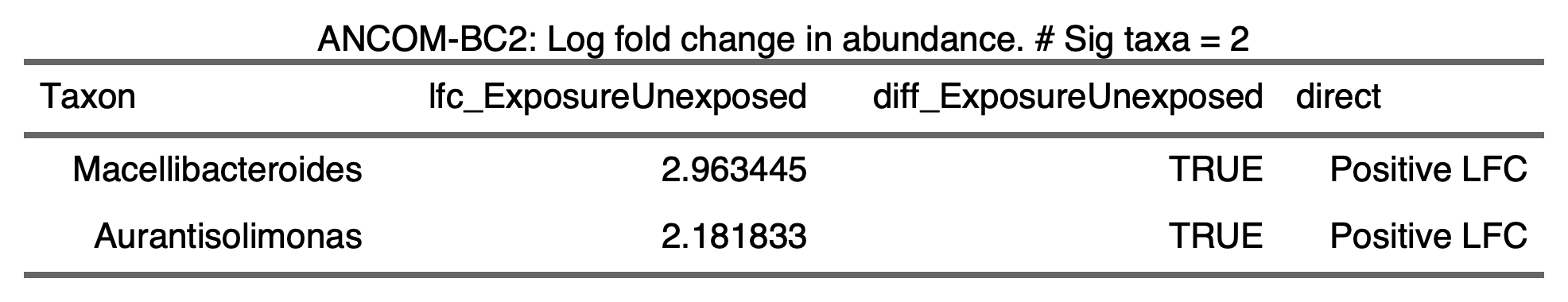
**

**3.5.3) Watts Diet**

**
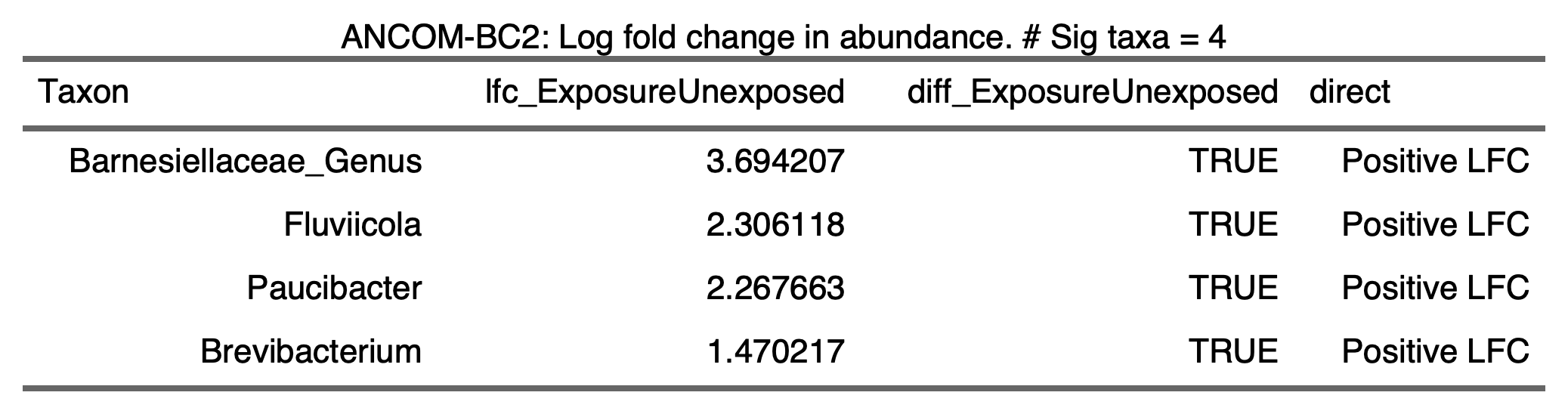
**

**3.5.4) ZIRC Diet**

**
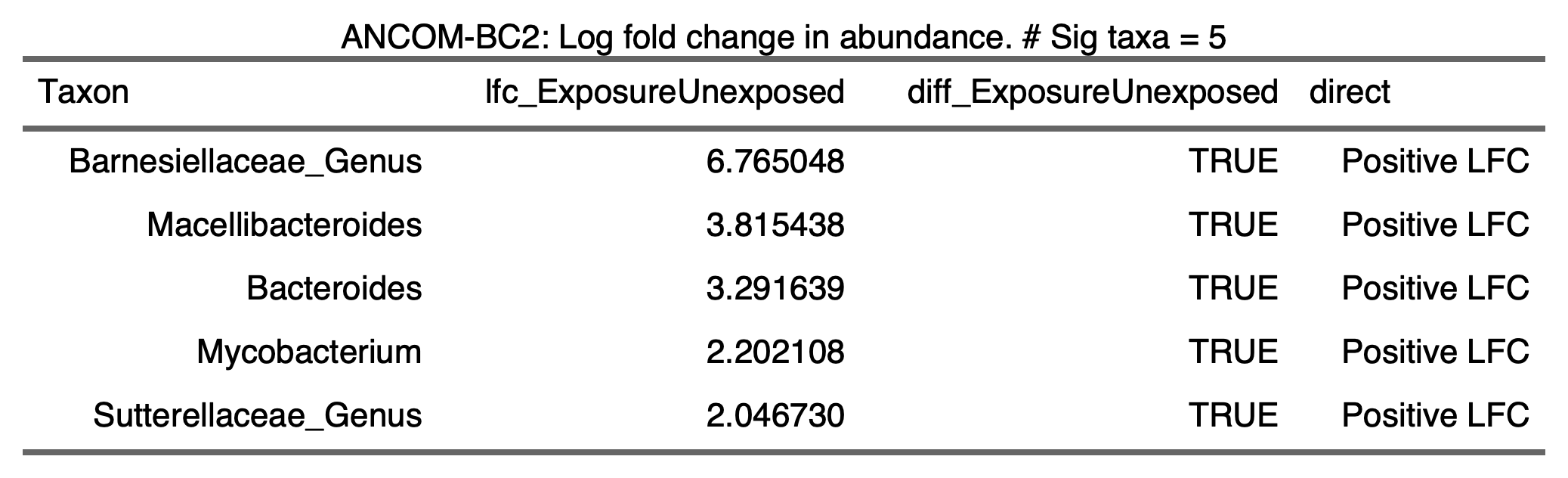
**

**3.5.5)**

**
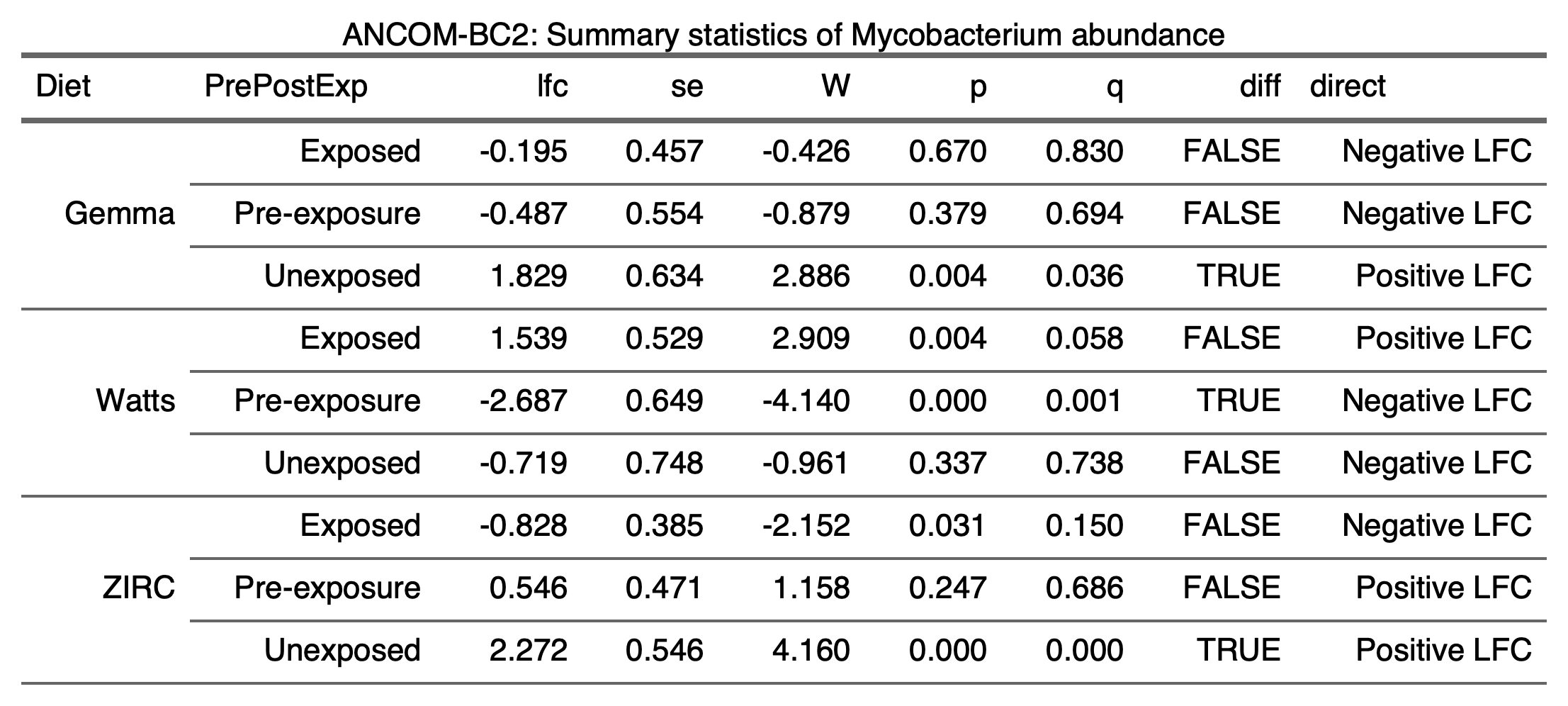
**

1. **Diet Nutrition and Formulations**

**4.1.1) Proximate and elemental analysis**

**
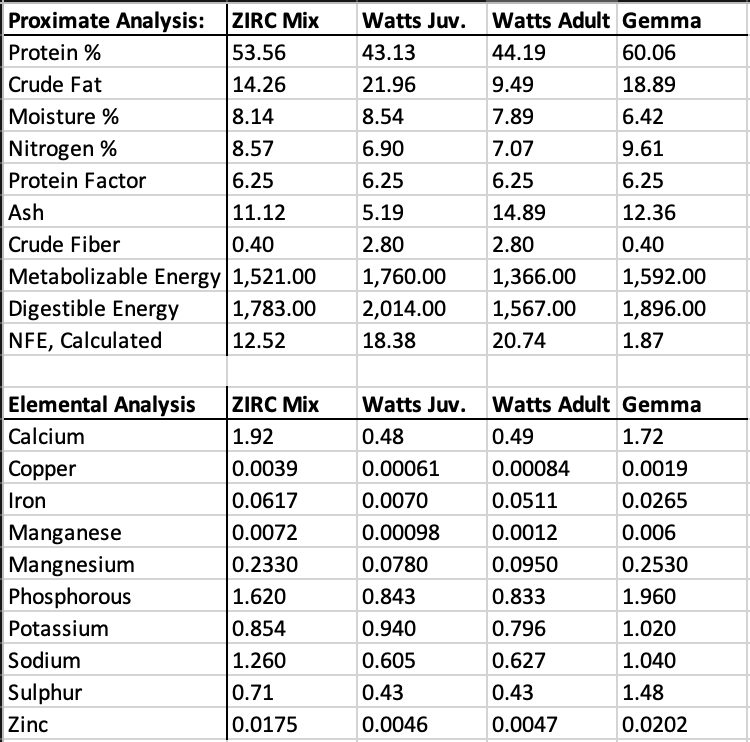
**
